# Absolute quantitative and base-resolution sequencing reveals comprehensive landscape of pseudouridine across the human transcriptome

**DOI:** 10.1038/s41592-024-02439-8

**Published:** 2024-09-30

**Authors:** Haiqi Xu, Linzhen Kong, Jingfei Cheng, Khatoun Al Moussawi, Xiufei Chen, Aleema Iqbal, Peter A. C. Wing, James M. Harris, Senko Tsukuda, Azman Embarc-Buh, Guifeng Wei, Alfredo Castello, Skirmantas Kriaucionis, Jane A. McKeating, Xin Lu, Chun-Xiao Song

**Affiliations:** 1 Ludwig Institute for Cancer Research, Nuffield Department of Medicine, University of Oxford, Oxford, UK; 2 Target Discovery Institute, Nuffield Department of Medicine, University of Oxford, Oxford, UK; 3 Chinese Academy of Medical Sciences Oxford Institute, Nuffield Department of Medicine, University of Oxford, Oxford, UK; 4 Nuffield Department of Medicine, University of Oxford, Oxford, UK; 5 MRC University of Glasgow Centre for Virus Research, Glasgow, UK; 6 Department of Biochemistry, University of Oxford, Oxford, UK

**Keywords:** RNA, RNA sequencing

## Abstract

Pseudouridine (Ψ) is one of the most abundant modifications in cellular RNA. However, its function remains elusive, mainly due to the lack of highly sensitive and accurate detection methods. Here, we introduced 2-bromoacrylamide-assisted cyclization sequencing (BACS), which enables Ψ-to-C transitions, for quantitative profiling of Ψ at single-base resolution. BACS allowed the precise identification of Ψ positions, especially in densely modified Ψ regions and consecutive uridine sequences. BACS detected all known Ψ sites in human rRNA and spliceosomal small nuclear RNAs and generated the quantitative Ψ map of human small nucleolar RNA and tRNA. Furthermore, BACS simultaneously detected adenosine-to-inosine editing sites and *N*^1^-methyladenosine. Depletion of pseudouridine synthases TRUB1, PUS7 and PUS1 elucidated their targets and sequence motifs. We further identified a highly abundant Ψ_114_ site in Epstein–Barr virus-encoded small RNA EBER2. Surprisingly, applying BACS to a panel of RNA viruses demonstrated the absence of Ψ in their viral transcripts or genomes, shedding light on differences in pseudouridylation across virus families.

## Abstract (web summary)

This study introduces a chemical method, BACS, that generates Ψ-to-C mutation signatures, allowing for sequencing and quantification of Ψ at single-base resolution.

## Main

Ψ is one of the most abundant posttranscriptional modifications in cellular RNA^1,2^. It is not only prevalent in various noncoding RNAs (ncRNAs), including ribosomal RNA (rRNA), small nuclear RNA (snRNA) and transfer RNA (tRNA)^3^, but also present in messenger RNA (mRNA)^4,5^. Ψ plays important roles in splicing, translation, RNA stability and RNA–protein interactions^6^. In eukaryotes, Ψ is installed by pseudouridine synthases (PUSs)^7^, which have been shown to associate with many diseases including cancer^6^. Therefore, establishing an accurate and sensitive method to detect Ψ is highly desirable.

Traditionally, the detection of Ψ has been reliant on the *N*-cyclohexyl-*N*′-(2-morpholinoethyl)carbodiimide methyl-*p*-toluenesulfonate (CMC) chemistry^8^. Since the stable *N*^3^–CMC adduct of Ψ blocks the base pairing and would lead to reverse transcription (RT) truncations, CMC chemistry has been widely applied to transcriptome-wide mapping of Ψ, as shown in Pseudo-seq^4^, Ψ-seq^5^ and CeU-seq^9^. However, CMC-based methods have low labeling efficiency and selectivity for Ψ and lack stoichiometry information, making it intrinsically difficult to distinguish between true Ψ signals and background noises.

Recently, RBS-seq reexamined the bisulfite (BS)-mediated conversion of Ψ, and found that the Ψ–BS adduct could lead to deletion signatures during RT^10,11^. BID-seq and similarly designed PRAISE further optimized BS treatment at near neutral pH to eliminate the side reaction on unmodified cytosine and enabled quantitative detection of Ψ^12,13^. However, due to the deletion signature, BS-based methods cannot determine the exact position of Ψ in consecutive uridine sequences or consecutive Ψ sites and struggle to detect densely modified Ψ sites.

To overcome these limitations, we developed BACS for direct, quantitative and base-resolution sequencing of Ψ based on new bromoacrylamide cyclization chemistry. BACS induces Ψ-to-C mutation signatures rather than truncation or deletion signatures during RT, thereby providing higher resolution and more accurate quantification of Ψ stoichiometry compared with CMC-based and BS-based methods. We applied BACS to various types of ncRNAs and mRNA to build a comprehensive map of Ψ across the human transcriptome. Besides Ψ mapping, BACS delivers simultaneous detection of adenosine-to-inosine (A-to-I) editing sites in mRNA and *N*^1^-methyladenosine (m^1^A) in tRNA. We further utilized BACS to elucidate genuine Ψ targets and sequence motifs of three key PUS enzymes (TRUB1, PUS7 and PUS1) in HeLa cells. Finally, we applied BACS to various RNA and DNA viruses to investigate the presence of Ψ in viral RNAs.

## Results

### Development of BACS

**Fig. 1:**
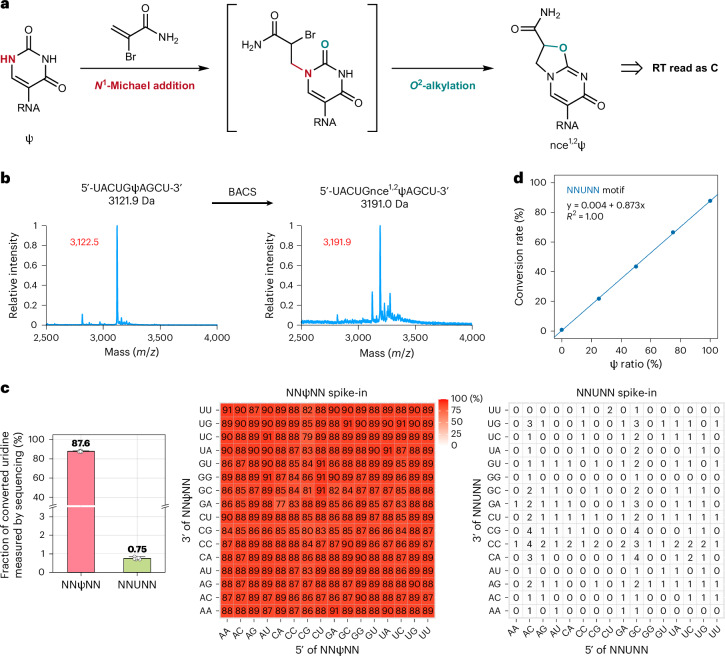
BACS achieved quantitative detection of Ψ through cyclization chemistry. **a**, Schematic overview of BACS reaction. **b**, MALDI characterization of BACS labeling of a 10mer Ψ-containing RNA oligonucleotide. Calculated mass is shown in black. Observed mass is shown in red. Data are representative of two independent experiments. **c**, Cumulative (left) and motif-dependent (middle and right) results of BACS conversion rates and false-positive rates on synthetic 30mer NNΨNN and NNUNN spike-in. Data are shown as means ± s.d. of six independent experiments (*n* = 6). **d**, BACS calibration curve for quantification of Ψ stoichiometry in NNUNN motif. Data are representative of two independent experiments. Source data

The most distinct difference between Ψ and uridine is the free *N*^1^ of Ψ, which is highly reactive toward Michael addition acceptors, such as acrylonitrile^14,15^, acrylamide^16^ and other acrylic compounds^17^. Selective labeling of Ψ by acrylonitrile has been widely used to distinguish Ψ from uridine in mass spectrometry^18^. However, a simple *N*^1^ adduct of Ψ with acrylic compounds would not induce mutation during RT. We envisioned that installing an α-halogen group would induce a tandem cyclization through intramolecular *O*^2^-alkylation and finally lead to Ψ-to-C mutation (Fig. 1a). We initially tested this chemistry on a short Ψ-containing oligonucleotide with 2-bromoacrylamide and analyzed the reaction product by matrix-assisted laser desorption/ionization mass spectrometry (MALDI). We found a 69-Da increase of mass values, indicating the formation of a cyclized product (carbamido-1, *O*^2^-ethano Ψ, nce^1,2^Ψ; Fig. 1b and Supplementary Fig. 1a). This reaction was further confirmed by ultra-high-performance liquid chromatography–tandem mass spectrometry (UHPLC–MS/MS; Supplementary Fig. 1b). To validate the mutation signature of nce^1,2^Ψ, we applied BACS to a 72mer RNA containing two Ψ sites (Supplementary Table 1). Through RT and next-generation sequencing, we obtained 86.6% U-to-C mutation rates on these two sites, while U-to-R (R = A or G) mutation rates were lower than 1% (Supplementary Fig. 1c). Therefore, we confirm that the U-to-C mutation rate can serve as the conversion rate of BACS.

To understand the sequence preference of BACS, we built libraries with synthetic 30mer RNA spike-in containing NNΨNN and NNUNN (N = A, C, G or U), respectively (Fig. 1c). After BACS, we observed an 87.6% conversion rate of Ψ and a 0.75% false-positive rate of uridine when accumulating all motifs. Among all 256 motifs, 230 showed a conversion rate higher than 85% and 254 displayed a conversion rate higher than 80%, suggesting the high efficiency of BACS chemistry. We observed a low false-positive rate (<1%) in most motifs (213 of 256 motifs), while certain motifs displayed slightly higher false-positive rates (3–4%). Nevertheless, BACS clearly showed higher conversion rates than BID-seq both in general and in specific motifs^12^. By mixing NNΨNN and NNUNN spike-in in different ratios, we generated excellent linear calibration curves for accurate quantification of Ψ modification levels (*R*^2^ = 1.00; Fig. 1d and Supplementary Fig. 1d).

### Validation of BACS on human rRNA

**Fig. 2:**
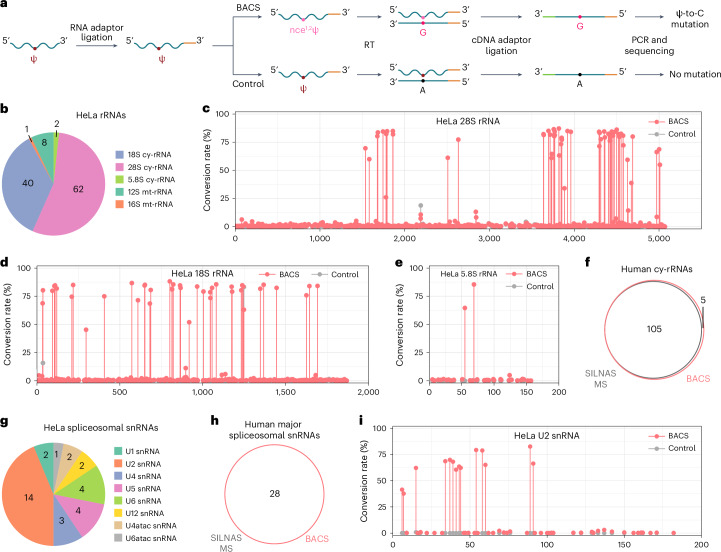
BACS detected known Ψ sites in human rRNA and spliceosomal snRNAs. **a**, Flowchart of BACS library construction. **b**, Numbers of Ψ sites identified in HeLa cy-rRNAs and mt-rRNAs. **c**–**e**, Conversion rates of BACS (pink) and control (gray) samples in HeLa 28S rRNA (**c**), 18S rRNA (**d**) and 5.8S rRNA (**e**), respectively. Data are presented as means of two independent experiments. **f**, Venn diagram illustrating the overlap of Ψ sites detected in human cy-rRNAs between BACS and SILNAS MS. **g**, Numbers of Ψ sites identified in HeLa spliceosomal snRNAs. **h**, Venn diagram illustrating the overlap of Ψ sites detected in human spliceosomal snRNAs between BACS and SILNAS MS. **i**, Conversion rates of BACS (pink) and control (gray) samples in HeLa U2 snRNA. Data are presented as means of two independent experiments. Source data

To evaluate the performance of BACS, we applied it to cytosolic rRNA (cy-rRNA) from cervical and nasopharyngeal cancer cell lines HeLa and C666-1, respectively (Fig. 2a). As expected, we observed high mutation rates only on Ψ, while other bases showed minimum mutation rates (Supplementary Fig. 2a,b). Using a 5% modification level cutoff, we detected 2, 40 and 62 Ψ sites in HeLa 5.8S, 18S and 28S rRNAs, respectively (Fig. 2b). Most of the sites displayed a high level of Ψ modification (>80%), consistent with reports that Ψ sites are highly modified in human cy-rRNA^19^ (Fig. 2c–e and Supplementary Fig. 2c–f). We also examined the raw signals of BACS, which revealed a strong correlation between two biological replicates (Pearson’s *r* = 1.00; Supplementary Fig. 2g). Compared with the reported SILNAS mass spectrometry (SILNAS MS) data, 103 of 105 known Ψ sites in human cy-rRNA (including one 2′-*O*-methylpseudouridine, Ψm site) were identified with high confidence^19^ (Fig. 2c–e and Supplementary Fig. 2c–f). However, Ψ_1136_ in 18S rRNA was not detected, possibly due to its low modification level in HeLa cells (4.5% by BACS; Supplementary Fig. 2c,d). Interestingly, we found a 16% U-to-C mutation rate of the known 18S rRNA Ψ_36_ site in control libraries, although the mutation rate increased to 80% after BACS treatment (Fig. 2d and Supplementary Fig. 2h). Similar results were obtained from BID-seq control libraries^12^, suggesting the presence of an uncharacterized single nucleotide polymorphism (SNP) site in HeLa cells. It is noteworthy that these two sites could be readily detected in C666-1 cells (Supplementary Fig. 2i). Therefore, BACS could detect all known Ψ sites in human cy-rRNAs (Fig. 2f). In addition, we detected a new Ψ_4938_ site in 28S rRNA from HeLa and C666-1 cells, located adjacent to the previously known Ψ_4937_ site (Supplementary Fig. 2e,f). The presence of Ψ_4938_ was supported by two public databases, both of which predicted that small nucleolar RNA (snoRNA) SNORA17B would be responsible for catalyzing this modification^20,21^. We further identified four new Ψ sites (Ψ_31_/Ψ_890_/Ψ_899_ in 18S rRNA and Ψ_1674_ in 28S rRNA) in cy-rRNAs from C666-1 cells (Supplementary Fig. 2i). It is important to note that while some Ψ or uridine modifications can induce intrinsic mutation signals (such as m^1^acp^3^Ψ_1248_ in 18S rRNA and m^3^U_4500_ in 28S rRNA), these can be filtered out by comparing results of BACS libraries with control libraries (Supplementary Fig. 2h). In addition to cy-rRNA, we also applied BACS to mitochondrial rRNA (mt-rRNA) and detected eight and one Ψ sites in 12S and 16S rRNAs, respectively (Fig. 2b). Among them, four sites have also been detected by Pseudo-seq^4^. In general, the modification level of Ψ sites in mt-rRNA was substantially lower than that in their cytosolic counterparts (Supplementary Fig. 2j).

As expected, BACS clearly outperformed BS-based methods in the following aspects^12,13^. Firstly, the U-to-C mutation signature enabled BACS to determine the exact position and number of Ψ sites in consecutive uridine sequences (adjacent to one or more uridines, for instance, Ψ_801_/Ψ_814_/Ψ_815_ in 18S rRNA, Ψ_1847_/Ψ_1849_ in 28S rRNA and Ψ_4323_/Ψ_4331_ in 28S rRNA; Supplementary Fig. 3a–c). The improved bioinformatics pipeline of BID-seq with realignment analysis (BID-pipe^22^) could not resolve ambiguity in two consecutive uridine sequences and may introduce artifacts in some cases (for example, Ψ_681_ in 18S rRNA, Ψ_1045_/Ψ_1046_ in 18S rRNA and Ψ_4549_ in 28S rRNA; Supplementary Fig. 3d–f). Secondly, more even conversion rates of Ψ sites were obtained over densely modified regions of rRNA using BACS compared with BS-based methods, indicating that BACS datasets would not be influenced by the density of pseudouridylation (Supplementary Fig. 3g,h). In particular, BACS successfully detected extremely dense Ψ sites in a narrow region (for example, Ψ_3737_/Ψ_3741_/Ψ_3743_/Ψ_3747_/Ψ_3749_ in 28S rRNA and Ψ_4263_/Ψ_4266_/Ψ_4269_ in 28S rRNA). Thirdly, when compared to SILNAS MS, BACS provided greatly improved accuracy in quantifying Ψ modification levels than BS-based methods (*r* = 0.90 for BACS, *r* = 0.37 for BID-seq, *r* = 0.48 for PRAISE; Supplementary Fig. 3i–k). These findings strongly indicate that quantifying Ψ using deletion signals, as done by BS-based methods, introduces inaccuracies into the analysis.

### BACS identified dense Ψ sites in human spliceosomal snRNAs

We next applied BACS to spliceosomal snRNAs from HeLa, C666-1, Raji and Elijah cells. In major spliceosomal snRNA species, we detected 2, 14, 3, 4 and 4 Ψ sites in U1, U2, U4, U5 and U6 snRNAs from HeLa cells, respectively, which is highly consistent with the latest SILNAS MS results^23^ (Fig. 2g). Only Ψ_59_ in U4 snRNA was not detected by BACS, as it is likely to be lowly modified in HeLa cells. However, this position was modified to higher levels in C666-1 and Elijah cells (Supplementary Fig. 4a). Consequently, we detected all known Ψ sites in human major spliceosomal snRNAs (Fig. 2h). It is noteworthy that BACS successfully mapped all 14 Ψ sites in human U2 snRNA, which has not been realized by any other high-throughput sequencing methods, further highlighting the superiority of BACS in detecting dense and consecutive Ψ sites (Fig. 2i). In minor spliceosomal snRNA species, we detected 2, 2 and 1 Ψ sites in U12, U4atac and U6atac snRNAs from HeLa cells, respectively, while no Ψ site was detected in U11 snRNA (Fig. 2g). Notably, we confirmed that there are two consecutive Ψ sites (Ψ_11_/Ψ_12_) rather than one Ψ_12_ site in U4atac snRNA^24^ (Supplementary Fig. 4b,c). Additionally, we detected highly conserved Ψ_247_ and Ψ_250_ sites in 7SK RNA^25^ and a differentially modified Ψ_211_ site in 7SL RNA^23^ (Supplementary Fig. 4a), while no high-confidence Ψ sites were detected in U7 snRNA, RNase P RNA, RNase MRP RNA, vault RNA and Y RNA (Supplementary Fig. 4d–h).

### BACS revealed the Ψ profile of human snoRNA

The Ψ profile of human snoRNA remains relatively unexplored. Ψ-seq^5^ and BID-seq^12^ only detected 11 and 39 Ψ sites in human snoRNA, respectively. In contrast, BACS detected 304 Ψ sites in snoRNA from HeLa cells, including 205, 67 and 32 Ψ sites in box C/D snoRNAs, box H/ACA snoRNAs and small Cajal body-specific RNAs, respectively, with a substantial number of highly modified sites (Supplementary Fig. 5a–c). SnoRNA Ψ sites detected through BACS largely covered those previously identified by Ψ-seq^5^ and BID-seq^12^, demonstrating increased sensitivity of BACS (Supplementary Fig. 5d,e). Furthermore, we observed that Ψ sites in box C/D snoRNAs displayed enrichment in the 5′-upstream regions of box D′ and the 3′-downstream regions of box C′, while Ψ sites in box H/ACA snoRNAs were enriched in the 5′-upstream regions of box H and ACA, implying a potential role for Ψ in mediating interactions between snoRNAs and their targets (Supplementary Fig. 6a,b). Indeed, a subset of Ψ sites identified in box C/D and box H/ACA snoRNAs was also located in the predicted guide regions, which was in accordance with the Ψ-seq results^5^ (Supplementary Fig. 6c,d).

In addition, human telomerase RNA component (TERC) shares similar characteristics with snoRNAs. Upon BACS treatment, we detected seven Ψ sites in TERC from HeLa cells, four of which were putative Ψ sites previously discovered through a CMC-based primer extension approach^26^ (Supplementary Fig. 5a,f). In particular, all three new Ψ sites (Ψ_38_/Ψ_100_/Ψ_155_), together with the known Ψ_161_ and Ψ_179_ sites, were found in the core domain of TERC, which may contribute to the stabilization of TERC structure, similarly to the scenario for Ψ_306_ and Ψ_307_ within the P6.1 loop^27^.

### A comprehensive Ψ map of human tRNA

**Fig. 3:**
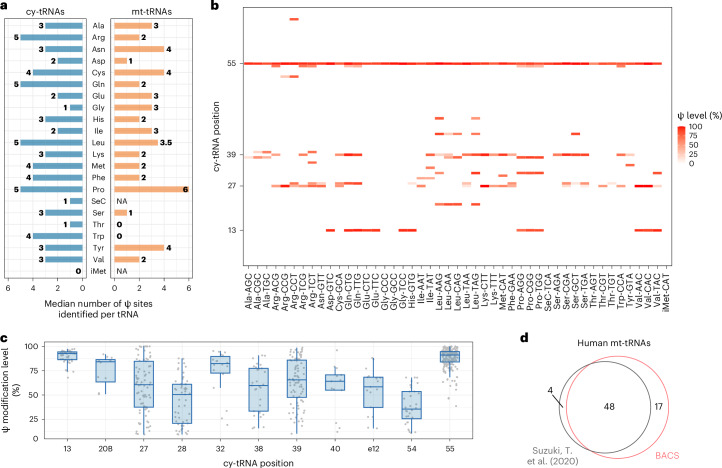
BACS unveiled the comprehensive Ψ profile of human tRNA. **a**, Median numbers of Ψ sites identified per tRNA in each cy-tRNA and mt-tRNA isotype from HeLa cells. NA, not applicable. **b**, Heat map showing the modification levels of high-confidence Ψ sites in HeLa cy-tRNAs. Only one representative tRNA isodecoder was presented for each isoacceptor family. **c**, Comparison of the modification levels of Ψ sites at selected positions of HeLa cy-tRNAs. Box plots visualize all Ψ sites at each position; boxes represent the 25th to 75th percentiles with a line at the median; whiskers correspond to 1.5 times the interquartile range (tRNA position: 13, *n* = 43; 20B, *n* = 12; 27, *n* = 81; 28, *n* = 53; 32, *n* = 18; 38, *n* = 29; 39, *n* = 86; 40, *n* = 13; e12, *n* = 17; 54, *n* = 32; 55, *n* = 185). **d**. Venn diagram illustrating the overlap of Ψ sites in human mt-tRNAs reported by BACS and a previously published dataset^31^. Source data

Ψ is one of the most fundamental and prevalent modifications in human tRNA^28^. However, CMC-based and BS-based methods failed to map Ψ in human cytosolic tRNAs (cy-tRNAs)^13,29^. We used BACS to generate the quantitative Ψ map of cy-tRNAs from HeLa cells with 609 high-confidence Ψ sites (Supplementary Fig. 7a). The number of Ψ sites identified per cy-tRNA varied among different isotypes (Fig. 3a). In cy-tRNAs, Ψ sites were predominantly located at highly conserved positions, including positions 13, 27–28, 38–40 and 55, while Ψ sites at other positions were limited to specific types of cy-tRNAs (Fig. 3b). An integrated view of the Ψ profile of human cy-tRNAs was summarized based on the canonical tRNA numbering system^30^ (Supplementary Fig. 7b). Notably, position 55 emerged as the most frequently and highly modified Ψ site in cy-tRNAs (Fig. 3c). Moreover, position 13 also displayed a high level of Ψ modification. In contrast, the modification levels of position 27–28 and position 38–40 showed considerable variations.

We also detected 54 Ψ sites in HeLa mitochondrial tRNAs (mt-tRNAs; Fig. 3a and Supplementary Fig. 7a). Applying BACS to other human cell lines, we observed notable differential Ψ modification on mt-tRNAs. For example, Ψ_55_ in mt-tRNA^Met^ was not characterized as a high-confidence site due to its low modification level in HeLa cells (3.4%), while it was readily detected in C666-1, Raji and Elijah cell lines (Supplementary Fig. 7c,d). In addition, C666-1 cell lines displayed an elevated Ψ level at position 66–68 compared to HeLa, Raji and Elijah cells (Supplementary Fig. 7d). We finally mapped a total of 65 Ψ sites in mt-tRNAs by merging the results of HeLa, C666-1, Raji and Elijah cells, which was highly consistent with the published dataset^31^ (Fig. 3d and Supplementary Table 13). Most of the BACS-only Ψ sites were located near the 3′-end of mt-tRNA, which could not be identified using CMC-based methods. Four Ψ sites in ref. ^31^ were not detected by BACS, including mt-tRNA^Gln^ Ψ_33_/Ψ_40_, mt-tRNA^His^ Ψ_35_ and mt-tRNA^Pro^ Ψ_67_. Overall, human mt-tRNAs were pseudouridylated to a lesser extent compared with cy-tRNAs (Fig. 3b,c and Supplementary Fig. 7c,d). In contrast, PRAISE detected only 34 Ψ sites in mt-tRNAs from HEK293T cells^13^ (Supplementary Fig. 8a). PRAISE encountered challenges in quantifying consecutive Ψ sites (8 of 34) and determining the precise position for a single Ψ site within multiple uridine contexts (13 of 34; Supplementary Fig. 8b–e). As a result, PRAISE achieved quantitative and single-base-resolution detection for only 13 Ψ sites in mt-tRNAs, revealing crucial limitations compared to BACS.

### Profiling and quantification of Ψ in HeLa mRNA

**Fig. 4:**
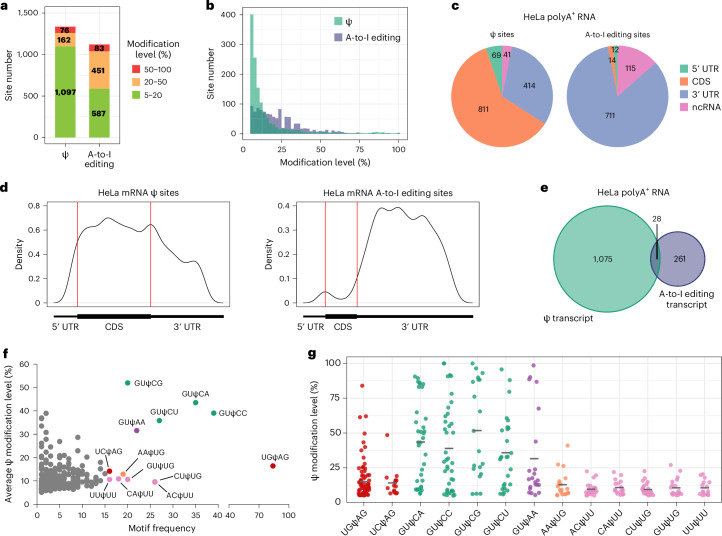
Simultaneous characterization of Ψ and A-to-I editing sites in HeLa mRNA. **a**, Numbers of Ψ and A-to-I editing sites with high (50–100%; red), medium (20–50%; yellow) and low (5–20%; green) modification levels identified in HeLa poly-A-tailed RNA. **b**, Modification level distribution of Ψ and A-to-I editing sites in HeLa poly-A-tailed RNA. **c**, Distribution of Ψ and A-to-I editing sites within different features of HeLa mRNA and ncRNA. **d**, Metagene profile of Ψ and A-to-I editing sites in HeLa mRNA. **e**, Venn diagram illustrating the overlap of transcripts possessing Ψ and A-to-I editing sites. **f**, Motif frequency of Ψ sites in HeLa mRNA. **g**, Modification level distributions of HeLa mRNA Ψ sites within selected motifs, with means indicated in each plot by a horizontal line. Motif: UGΨAG, *n* = 84; UCΨAG, *n* = 16; GUΨCA, *n* = 35; GUΨCC, *n* = 39; GUΨCG, *n* = 20; GUΨCU, *n* = 27; GUΨAA, *n* = 22; AAΨUG, *n* = 19; ACΨUU, *n* = 26; CAΨUU, *n* = 18; CUΨUG, *n* = 26; GUΨUG, *n* = 20; UUΨUU, *n* = 16. CDS, coding sequence. Source data

After successfully applying BACS to various types of ncRNAs, we extended its usage to map and quantify Ψ modifications in HeLa mRNA. Given the low stoichiometry of Ψ modification in poly-A-tailed RNA, we applied in vitro transcribed poly-A-tailed RNA (IVT RNA) from HeLa cells as a modification-free control to help with Ψ calling^32^ (Supplementary Fig. 9a). We detected a total of 1,335 Ψ sites in HeLa poly-A-tailed RNA (Fig. 4a). In contrast to the aforementioned ncRNAs, the Ψ modification level in poly-A-tailed RNA was considerably lower (Supplementary Fig. 9b), with the majority exhibiting low modification levels (<20%) and only a limited number of Ψ sites displaying high levels of modification (>50%; Fig. 4a,b). Among the 1,335 Ψ sites, 1,294 and 41 were located in mRNA and ncRNA (excluding rRNA, snRNA, snoRNA and tRNA), respectively (Fig. 4c). Within mRNA, Ψ was enriched in the coding sequence and 3′-untranslated region (3′ UTR), consistent with previous findings^9,12,13^ (Fig. 4c,d). The 1,335 Ψ sites were located across 1,103 poly-A-tailed RNA transcripts, with the majority carrying only one Ψ site (Fig. 4e). Gene Ontology (GO) analysis revealed that Ψ-modified mRNA was enriched in functions such as translation and regulation of apoptotic process (Supplementary Fig. 9c). Importantly, BACS could simultaneously provide mRNA expression levels while mapping Ψ, which showed strong correlation with control libraries (Pearson’s *r* = 0.99–1.00), suggesting minimal RNA degradation induced by BACS (Supplementary Fig. 9d).

Next, we analyzed the sequence contexts of Ψ in HeLa mRNA. First, the majority of Ψ sites (55.3%) were located in consecutive uridine sequences, which could not be precisely determined through BS-based methods^12,13^ (Supplementary Fig. 10a). Benefiting from the high resolution of BACS, we found that Ψ was predominantly enriched in USΨAG (S = C or G) and GUΨCN (N = A, C, G or U) motifs, corresponding to the previously identified PUS7 and TRUB1 motifs, respectively^33^ (Fig. 4f). In addition, we also observed that Ψ tends to be enriched in those motifs containing multiple consecutive uridines, such as CUΨUG, ACΨUU and UUΨUU. Among all motifs, GUΨCN exhibited a relatively high modification level (Fig. 4g). Furthermore, we analyzed the codon preference of Ψ in mRNA. As expected, Ψ was enriched in those codons containing consecutive uridines, such as UUY (Y = C or U), UUG, AUU and GUU, which encoded phenylalanine (Phe), leucine (Leu), isoleucine (Ile) and valine (Val), respectively (Supplementary Fig. 10b,c). Within codons, Ψ was mainly located in the second position (Supplementary Fig. 10d). Moreover, we observed one Ψ site positioned in the start codon (AUG), while two sites were found in the stop codon (UAG), which may promote stop codon readthrough according to previous research^12,34^.

To further evaluate the performance of BACS, we compared our identified mRNA Ψ sites with published datasets. We first compared BACS with a recent dataset that consolidated three CMC-based methods^33^. Remarkably, BACS accurately identified 61 of 70 Ψ sites (87.1%) listed in the ‘highest confidence’ category (Supplementary Fig. 11a). However, a strong overlap between BACS and the ‘high-confidence’ list was achieved only when considering Ψ sites consistently detected across multiple samples (>8; 177 of 320 Ψ sites, 55.3%), indicating the considerable variance between different CMC-based datasets (Supplementary Fig. 11b,c). We further compared BACS with two recently developed BS-based methods. Compared to CMC-based approaches, BACS demonstrated a better overlap with BID-seq results^12^ (230 of 575 Ψ sites, 40.0%; Supplementary Fig. 11d). Most of the sites exclusive to the BID-seq dataset displayed low modification levels in our BACS libraries, possibly due to the inaccurate quantification of BID-seq (Supplementary Fig. 11e). When compared with PRAISE^13^, 651 of 1,995 Ψ sites (32.6%) showed an overlap with BACS results (Supplementary Fig. 11f). Similarly, the majority of PRAISE-only Ψ sites were lowly modified in our dataset (Supplementary Fig. 11g). Regarding the 7 Ψ sites identified in mitochondrial mRNAs (mt-mRNAs) by BACS, 4, 2 and 4 of them have also been detected by Pseudo-seq^4^, BID-seq^12^ and PRAISE^13^, respectively.

### Simultaneous profiling of A-to-I editing sites and m^1^A

In addition to Ψ mapping, BACS enabled the simultaneous detection of A-to-I editing sites in a similar way with ICE-seq^35^ (Supplementary Fig. 9a). We identified 1,121 A-to-I editing sites in HeLa poly-A-tailed RNA, with a mean modification level of 20% (Fig. 4a,b). In stark contrast to Ψ, most of A-to-I editing sites were resident in the *Alu* elements (Supplementary Fig. 9e). We further annotated 737 and 115 A-to-I editing sites to mRNA and ncRNAs, respectively (Fig. 4c). Within mRNA, the A-to-I editing sites were predominantly enriched in 3′ UTR, consistent with previous findings^36^ (Fig. 4d). Interestingly, mRNA transcripts that carry A-to-I editing sites did not overlap with those possessing Ψ, suggesting distinct roles of A-to-I editing and pseudouridylation in mRNA processing (Fig. 4e).

Similarly to RBS-seq^10^, BACS induces Dimroth rearrangement of m^1^A to *N*^6^-methyladenosine (m^6^A) and could potentially detect m^1^A together with Ψ (ref. ^37^; Supplementary Fig. 12a). As expected, we observed a marked reduction of m^1^A mutation signals at tRNA position 58 (for cy-tRNAs) and 9 (for mt-tRNAs) after BACS treatment (Supplementary Fig. 12b,c). These results highlight the value of BACS to detect multiple modifications simultaneously.

### BACS uncovered new PUS targets in HeLa cells

**Fig. 5:**
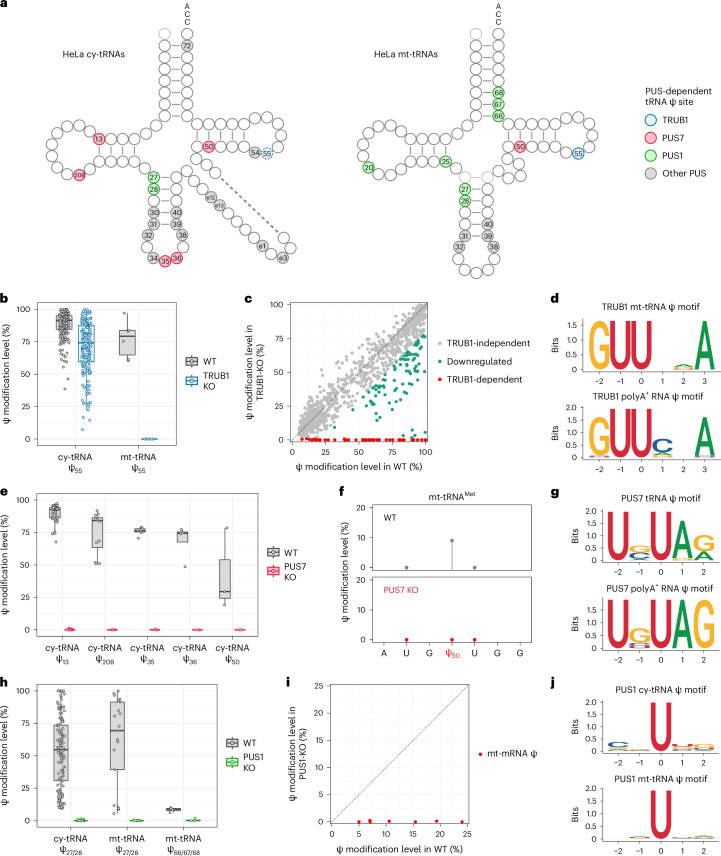
PUS-dependent Ψ landscape across the HeLa transcriptome. **a**, Integrated view of the PUS-dependent Ψ profiles of HeLa cy-tRNAs and mt-tRNAs. Ψ_55_ in HeLa cy-tRNAs is partially dependent on TRUB1, which is labeled by the dashed line. **b**, Comparison of the modification levels of Ψ_55_ in HeLa cy-tRNAs and mt-tRNAs upon TRUB1 depletion. Box plots visualize all Ψ sites at each position; boxes represent the 25th to 75th percentiles with a line at the median; whiskers correspond to 1.5 times the interquartile range (cy-tRNA Ψ_55_, *n* = 180; mt-tRNA Ψ_55_, *n* = 6). **c**, Scatterplot illustrating all TRUB1-dependant Ψ sites across the HeLa transcriptome. **d**, Sequence motifs of TRUB1-dependent Ψ sites in mt-tRNAs and poly-A-tailed RNA. **e**, Comparison of the modification levels of Ψ sites at selected positions of HeLa cy-tRNAs upon PUS7 depletion. Box plots visualize all Ψ sites at each position; boxes represent the 25th to 75th percentiles with a line at the median; whiskers correspond to 1.5 times the interquartile range (cy-tRNA: Ψ_13_, *n* = 43; Ψ_20B_, *n* = 12; Ψ_35_, *n* = 7; Ψ_36_, *n* = 4; Ψ_50_, *n* = 3). **f**, Comparison of the modification levels of Ψ_50_ in mt-tRNA^Met^ between wild-type (WT) and PUS7-KO cell lines. **g**, Sequence motifs of PUS7-dependent Ψ sites in tRNAs and poly-A-tailed RNA. **h**, Comparison of the modification levels of Ψ sites at selected positions of HeLa cy-tRNAs and mt-tRNAs upon PUS1 depletion. Box plots visualize all Ψ sites at each position; boxes represent the 25th to 75th percentiles with a line at the median; whiskers correspond to 1.5 times the interquartile range (cy-tRNA: Ψ_27/28_, *n* = 130; mt-tRNA: Ψ_27/28_, *n* = 21; Ψ_66/67/68_, *n* = 4). **i**, Scatterplot illustrating all PUS1-dependant Ψ sites in HeLa mt-mRNAs. **j**, Sequence motifs of PUS1-dependent Ψ sites in cy-tRNAs and mt-tRNAs. Source data

To elucidate the PUS-dependent Ψ profile in the HeLa transcriptome, we generated individual knockouts for three key PUS enzymes: TRUB1, PUS7 and PUS1 (Fig. 5a). In contrast to the conventional view that TRUB1 was the sole PUS enzyme responsible for cy-tRNA Ψ_55_ (ref. ^38^), depletion of TRUB1 did not eradicate Ψ_55_ in human cy-tRNAs, suggesting redundancy of PUS enzymes for this position (Fig. 5b,c). Surprisingly, all Ψ_55_ sites in mt-tRNA were eliminated upon TRUB1 depletion, challenging another belief that TRUB2 was solely responsible for mt-tRNA Ψ_55_ (ref. ^39^; Fig. 5b,c). We further discovered that the TRUB1 motif would extend beyond the recognized GUΨCNA (N = A, C, G or U) motif^5,33^, since it could modify GUΨUAA in mt-tRNA^Asn^, GUΨGUA in mt-tRNA^Glu^ and GUΨAAA in mt-tRNA^Pro^ with high efficiency (Fig. 5d and Supplementary Fig. 13a–c). We observed similar results in poly-A-tailed RNA, confirming that TRUB1 could edit GUΨANA, GUΨGNA and GUΨUNA motifs (Fig. 5d). Therefore, we demonstrate that the TRUB1 motif can be extended from GUΨCNA to GUΨNNA.

PUS7-knockout (KO) HeLa cells allowed us to reveal its role in the formation of Ψ_20B_, Ψ_36_ and Ψ_50_ in cy-tRNAs, in addition to its previously known targets Ψ_13_ and Ψ_35_ (ref. ^40^; Fig. 5e and Supplementary Fig. 13d). Moreover, our study uncovers new PUS7 activity in mitochondria by catalyzing the modification of Ψ_50_ in mt-tRNA^Met^ (Fig. 5f). The majority of PUS7 targets in tRNA displayed the conserved UVΨAR (V = A, C or G; R = A or G) motif (Fig. 5g). However, PUS7 displayed comparable activity within the UGΨGG motif (cy-tRNA^Arg(CTT)^ Ψ_50_) and relatively low activity within the UGΨUG motif (mt-tRNA^Met^ Ψ_50_; Fig. 5f,g and Supplementary Fig. 13e). In poly-A-tailed RNA, PUS7 mainly catalyzed the pseudouridylation within the UBΨAG (B = C, G or U) motif (Fig. 5g). Collectively, the true PUS7 consensus motif would be UNΨAR (N = A, C, G or U; R = A or G) and UGΨKG (K = G or U), which was less strict than previously considered UGΨAR (R = A or G) motif^4,41^.

Finally, PUS1 depletion resulted in the complete loss of Ψ_27/28_ in human cy-tRNAs (Fig. 5h and Supplementary Fig. 13f). In mt-tRNAs, PUS1 not only catalyzed the modification of Ψ_27/28_ but also induced the formation of Ψ_66/67/68_, consistent with the findings on yeast and mouse PUS1 homologs^42^ (Fig. 5h and Supplementary Fig. 13f). We also observed noncanonical activity of PUS1, exemplified by Ψ_25_ in mt-tRNA^Asn^ and Ψ_20_ in mt-tRNA^Leu(UUR)^ (Supplementary Fig. 13g,h). Additionally, PUS1 was found to catalyze all seven Ψ sites identified in mt-mRNAs (Fig. 5i). These results show that PUS1 is the major PUS enzyme in mitochondria with diverse functions. PUS1-dependent Ψ sites did not show any sequence motifs, consistent with an earlier report that its activity is dependent on RNA structure^41^ (Fig. 5j).

Mimicry of tRNA has been considered as a general way for mRNA pseudouridylation^5,33^. Although TRUB1, PUS7 and PUS1 could edit both tRNAs and poly-A-tailed RNA, the Ψ targets in poly-A-tailed RNA were substantially less modified than their counterparts in tRNAs (Supplementary Fig. 13i). These results suggest that mRNA may not be the primary substrate of these stand-alone PUS enzymes, consistent with our data showing that the majority of mRNA Ψ sites were modified to a low level.

### Mapping of Ψ in viral RNAs

**Fig. 6:**
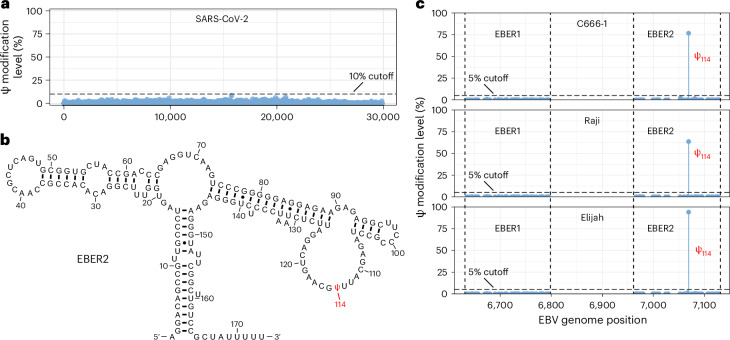
Investigation of Ψ modification in viral RNAs. **a**, Ψ modification levels in SARS-CoV-2 viral RNA. **b**, Canonical EBER2 structure, with the Ψ_114_ site labeled accordingly. **c**, Ψ modification levels in EBER1 and EBER2 from C666-1, Raji and Elijah cell lines. Source data

It has been widely accepted that Ψ-modified RNAs can suppress innate immune responses and may influence the mRNA vaccine design^43^. Previous studies have reported the presence of Ψ in severe acute respiratory syndrome coronavirus 2 (SARS-CoV-2) by Nanopore sequencing^44,45^; however, it has not been thoroughly confirmed with the latest sequencing technologies. Therefore, we applied BACS to five RNA viruses, including SARS-CoV-2, hepatitis C virus (HCV), Zika virus (ZIKV), hepatitis delta virus (HDV) and Sindbis virus (SINV). Surprisingly, we did not detect any high-confidence Ψ sites in the five RNA viruses (Fig. 6a and Supplementary Fig. 14). Importantly, we confirmed robust viral infection in our model systems, to ensure a high abundance of viral RNAs concomitant with high depth of coverages (Supplementary Table 3). These results suggest that Ψ is not directly involved in the modification of these RNA viruses.

In addition to these RNA viruses, we also applied BACS to human cell lines infected by Epstein–Barr virus (EBV), a DNA virus that encodes two highly expressed ncRNAs, EBER1 and EBER2. A previous study using HydraPsi-seq^46^ and CMC-based primer extension methods reported one lowly modified Ψ_160_ site in EBER2 (ref. ^47^; Fig. 6b). However, we detected one highly modified Ψ_114_ site in EBER2, while no Ψ site was identified in EBER1, indicating the previous results were likely caused by high backgrounds from hydrazine and CMC chemistry (Fig. 6b,c). The EBER2 Ψ_114_ site was conserved across all EBV strains and host cell lines tested (Fig. 6c). To validate this Ψ site, we applied modified BID-seq (Methods) to EBV-encoded ncRNAs from Raji and Elijah cell lines. Indeed, we detected a potential Ψ site within the three consecutive uridines (U_112_–U_114_) in EBER2, while no Ψ site was found in EBER1 (Supplementary Fig. 15a). However, BS-based methods could not determine the exact position of this site due to the limitations of deletion signals, which further strengthens the advantages of BACS (Supplementary Fig. 15b). Taken together, these results suggest different ways of utilizing Ψ between virus families and further highlight BACS as a highly specific method.

## Discussion

In this study, we report the development of BACS for quantitative and base-resolution sequencing of Ψ. BACS is based on new bromoacrylamide cyclization chemistry and induces Ψ-to-C mutation signatures rather than truncation or deletion signatures, allowing for accurate quantification of Ψ stoichiometry and sequencing of Ψ at absolute single-base resolution. Importantly, BACS overcomes the inherent limitations of BS-based methods in three crucial aspects: (1) it facilitates the precise determination of Ψ sites located adjacent to one or more uridines; (2) it enhances the detection of densely modified Ψ sites with higher accuracy and sensitivity; and (3) it offers much more accurate quantification of Ψ in all sequence contexts. These advances make BACS a valuable tool for studying Ψ modifications in cellular RNAs, as it can provide a more comprehensive and accurate picture of the Ψ landscape across various RNA species.

Using BACS, we successfully detected all known Ψ sites in human rRNA and spliceosomal snRNAs and generated the quantitative Ψ map of human snoRNA and tRNA. We further applied BACS to HeLa mRNA and revealed a rather low level of pseudouridylation. However, recent research has highlighted that Ψ could be much more abundant in pre-mRNA^48^. In the future, BACS could be extended to investigate the pseudouridylation of nascent RNA. Moreover, by genetic depletion of PUS enzymes, we identified new targets of TRUB1, PUS7 and PUS1 in HeLa cells. The absolute single-base resolution of BACS enabled us to extend the sequence motifs of TRUB1 and PUS7. Indeed, BACS could serve as a valuable tool for studying PUS knockout cells to elucidate the properties and functions of the other PUS enzymes. Finally, we redefined the Ψ landscape of EBV-encoded ncRNAs EBER1 and EBER2, and several human RNA viruses (SARS-CoV-2, HCV, ZIKV, HDV and SINV), showing the importance of having a highly sensitive and specific method like BACS.

In light of these potential applications, we anticipate BACS to be widely adopted as the new standard to advance our understanding of Ψ modifications and their functional implications in diverse biological processes.

## Methods

### Preparation of model RNA

Regular and Ψ-labeled 10mer RNA oligonucleotides and 30mer spike-ins were purchased from Integrated DNA Technologies. The 72mer Ψ-containing model RNA used for mutation analysis and the 1.8-kb 10% Ψ-modified RNA used for UHPLC–MS/MS were prepared by T7 in vitro transcription using HiScribe T7 High Yield RNA Synthesis Kit (NEB) and Pseudo-UTP (Jena Bioscience) according to the manufacturer’s protocol. The DNA template was removed by adding 2 μl Turbo DNase (Invitrogen) to the reaction and incubating at 37 °C for 30 min. The products were finally purified with Monarch RNA Cleanup Kit (NEB). Sequences of RNA oligonucleotides can be found in Supplementary Table 1.

### Mass spectrometry analysis of short oligonucleotides

MALDI was performed on a Voyager-DE Biospectrometry Workstation (Applied Biosystems) with 2′,4′,6′-trihydroxyacetophenone as matrix. All the oligonucleotides were analyzed in positive mode.

### Quantification of Ψ level by UHPLC–MS/MS

The untreated and treated RNA were digested into nucleosides by Nucleoside Digestion Mix (NEB) in a 50 µl solution according to the manufacturer’s protocol. After filtering with Amicon Ultra-0.5 ml centrifugal filters (molecular weight cutoff of 3 kDa; Millipore), the digested samples were subjected to UHPLC–MS/MS analysis as described before^49^. The 1290 Infinity LC System (Agilent) was equipped with a ZORBAX RRHD SB-C18 column (2.1 × 150 mm, 1.8 μm, Agilent) coupled to a 6495B Triple Quadrupole Mass Spectrometer (Agilent). The ions were monitored in positive mode with mass transitions of *m/z* 245 to 125 (Ψ+H) and *m/z* 245 to 113 (rU+H; Supplementary Table 2). Concentrations of nucleosides in RNA samples were deduced by fitting the signal peak areas into the standard curves.

### Cell culture

HeLa cells (gifted from P. J. Ratcliffe, University of Oxford; originally obtained from the American Type Culture Collection (ATCC), CCL-2) were cultured in DMEM medium (Gibco) supplemented with 10% (vol/vol) FBS (Gibco) and 1% penicillin–streptomycin (Gibco) at 37 °C with 5% CO_2_. For isolation of RNA, cells were harvested by centrifugation for 5 min at 1,000*g* at room temperature.

C666-1, Raji and Elijah cells were grown in RPMI 1640 medium (Thermo), complemented with 10% (vol/vol) FBS (Biosera), 2 mM l-glutamine (Thermo) and 100 units per ml penicillin and 100 µg ml^−1^ streptomycin (Thermo). Cells were grown in a humidified incubator at 37 °C with 5% CO_2_. The C666-1 cell line was gifted from C. Dawson (University of Warwick). Raji and Elijah cell lines were gifted from P. Farrell (Imperial College London). Cell lines were tested for mycoplasma monthly using MycoAlert Kit (Lonza) and were sent for authentication by Eurofins genomics.

### Generation of CRISPR knockout cell lines

TRUB1-KO, PUS7-KO and PUS1-KO HeLa cells were generated using CRISPR–Cas9 technology (Supplementary Figs. 16 and 17). Briefly, single guide RNA (sgRNA) sequences were cloned into PX459 plasmids^50^. Transfection was performed using Lipofectamine 3000 Transfection Reagent (Invitrogen) following the manufacturer’s protocol. Cells were then selected by 2 µg ml^−1^ puromycin (Thermo). Serial dilution was performed to achieve clonal isolation. Finally, clones were expanded and picked for western blot validation with TRUB1 antibody (Proteintech, 12520-1-AP; 1:1,000 dilution), PUS7 antibody (Abcam, ab226257; 1:10,000 dilution) and PUS1 antibody (Proteintech, 11512-1-AP; 1:1,000 dilution). The sgRNA sequences were listed as follows:

TRUB1: 5′-CACGGCGAACACGCCGCTCAAGG-3′;

PUS7-sgRNA1: 5′-TTAATATTGAAACCCCGCTCTGG-3′;

PUS7-sgRNA2: 5′-TCGGAATGCAGTCTAACCAAAGG-3′;

PUS1: 5′-AATACAGCCTGACCGGACGAGGG-3′.

### RNA isolation

Total RNA was isolated using TRIzol (Invitrogen) and Direct-zol RNA Miniprep Plus (Zymo Research) according to the manufacturer’s protocol. Ribo^−^ RNA was isolated using RiboMinus Eukaryote System v2 (Invitrogen) according to the manufacturer’s protocol. Poly-A^+^ RNA was isolated by two rounds of poly-A-tailed selection using Dynabeads mRNA DIRECT Purification Kit (Invitrogen) according to the manufacturer’s protocol. To remove genomic DNA contamination, RNA was then treated with Turbo DNase and purified by Zymo-IC Column with RNA binding buffer.

### Viral infection and RNA isolation

#### SARS-CoV-2

Viral stocks were propagated as previously reported^51^. Briefly, Vero-TMPRSS2 cells (NIBSC, 100978) were infected with SARS-CoV-2 Victoria 02/20 strain at a multiplicity of infection (MOI) of 0.003 and incubated for 48–72 h until a visible cytopathic effect was observed. Viral titers were then determined by plaque assay from clarified supernatants. For sequencing, Calu-3 cells (gifted from N. Zitzmann, University of Oxford; originally obtained from the ATCC, HTB-55) were infected at an MOI of 1 for 1 h at 37 °C. The inoculum was then removed, cells washed thrice with PBS and incubated in Advanced DMEM with 10% FCS at 37 °C for 24 h. Total RNA was extracted using the RNeasy Mini Kit (Qiagen) and infection confirmed plaque assay and RT–qPCR using primers for the viral N gene: forward 5′-CACATTGGCACCCGCAATC-3′, reverse 5′-GAGGAACGAGAAGAGGCTTG-3′.

#### HCV and ZIKV

As previously reported^52^, the ZIKV MP1751 strain was propagated in Vero cells (ATCC, CCL-81) and concentrated with 8% polyethylene glycol (PEG) in NTE buffer. For infection, Huh-7.5 cells (gifted from C. Rice, Rockefeller University) were inoculated with either HCV or ZIKV (MOI 1) for 180 min, before extensive washing with PBS. The medium was replaced and infected cells were cultured for 72 h before lysing in RLT buffer. RNAs were extracted using RNeasy Mini Kit (Qiagen) and infection confirmed using RT–qPCR quantification of viral RNAs using specific primers pairs. HCV: forward 5′-TCCCGGGAGAGCCATAGTG-3′, reverse 5′-TCCAAGAAAGGACCCAGTC-3′; ZIKV: forward 5′-TCGTTGCCCAACACAAG-3′, reverse 5′-CCACTAATGTTCTTTTGCAGACAT-3′; and RPLP0: forward 5′-GCAATGTTGCCAGTGTCTG-3′, reverse 5′-GCCTTGACCTTTTCAGCAA-3′.

#### HDV

HDV inoculum was prepared by concentrating the culture supernatant of Huh-7 cells (gifted from A. Patel, University of Glasgow) transfected with pSVL(D3) and pT7HB2.7 plasmids as previously reported^53^. HepG2-NTCP cells (gifted from S. Urban, University of Heidelberg) were differentiated with 2.5% dimethylsulfoxide (DMSO)-containing culture medium for 72 h before viral infection, before inoculation with HDV (MOI 50) in the presence of 4% PEG 8000 and 2.5% DMSO for 24 h. After 24 h, cells were washed with PBS and cultured for an additional 5 days in the presence of 2.5% DMSO. Cells were lysed in RLT buffer, and total cellular RNA was purified using RNeasy Mini Kit (Qiagen) and RNase-free DNase Set (Qiagen). Infection was confirmed by RT–qPCR using specific primers to detect HDV transcripts.

#### SINV

SINV was produced from pT7-SVwt plasmid^54^ that was first linearized with XhoI and purified to use it as a template for in vitro RNA transcription with HiScribe T7 ARCA mRNA kit (NEB). Transcribed viral RNA was transfected into BHK-21 cells (ATCC, CCL-10) using Lipofectamine 3000 reagent (Invitrogen) according to the manufacturer’s instruction. Viruses were collected from the supernatant 24 h later and cleared by centrifugation at 2,000 rpm for 5 min followed by filtration with 0.45-μm PVDF syringe filter units (Merck) and stored at −80 °C. Cleared supernatants were titrated by plaque assay.

Poly-A^+^ RNA purification was performed based on the previously described protocols^55,56^, with the following alterations: A549 cells (ATCC, CCL-185) were seeded in two 10-cm dishes in DMEM 10% FBS 24 h before infection to reach 80% confluence. Next, cells were either mock-infected or infected using 0.1 MOI of SINV for 1 h in serum-free DMEM at 37 °C, followed by the replacement of the medium with DMEM supplemented with 2% FBS and incubated for 18 h. Cells were lysed with 1 ml of lysis buffer (20 mM Tris-HCl pH 7.5, 500 mM lithium chloride, 0.5% (wt/vol) lithium dodecyl sulfate, 1 mM EDTA, 0.1% IGEPAL (NP-40) and 5 mM dithiothreitol (DTT)). Lysates were homogenized by passing the lysate at high speed through a 5-ml syringe with a 27-gauge needle, repeating this process until the lysate was fully homogeneous. Then, the whole lysate was incubated with pre-equilibrated oligo(dT)25 magnetic beads (NEB) for 1 h at 4 °C with gentle rotation. Beads were collected in the magnet and washed twice with 2 ml of buffer 1 (20 mM Tris-HCl pH 7.5, 500 mM lithium chloride, 0.1% (wt/vol) lithium dodecyl sulfate, 1 mM EDTA, 0.1% IGEPAL and 5 mM DTT) for 5 min at 4 °C with gentle rotation, followed by two washes with buffer 2 (20 mM Tris-HCl pH 7.5, 500 mM lithium chloride, 1 mM EDTA, 0.01% IGEPAL and 5 mM DTT). Beads were then washed twice with 2 ml of buffer 3 (20 mM Tris-HCl pH 7.5, 200 mM lithium chloride, 1 mM EDTA and 5 mM DTT) at room temperature. Finally, beads were resuspended in 50 µl of elution buffer and incubated for 7 min at 55 °C with agitation. Eluates were stored at −80 °C. Approximately 5 µg of poly-A^+^ RNAs was used for further rRNA removal using the Ribo-Zero kit from the TruSeq Stranded Total RNA LT Kit (Illumina). Subsequently, RNAclean XP (Beckman Coulter) purification was conducted, and RNAs were finally eluted into 10–15 µl of nuclease-free water. The concentration and quality of the RNA were assessed using Qubit and RNA Bioanalyzer.

#### Preparation of IVT RNA control

A total of 100 ng poly-A^+^ RNA was annealed with 2 μl of 10 μM Oligo(dT)_30_VN primer (5′-TTTTTTTTTTTTTTTTTTTTTTTTTTTTTTVN-3′) and 2 μl of 10 mM dNTP mix (NEB) in 12 μl solution, incubated at 70 °C for 5 min and held at 4 °C. Next, 5 μl 4× Template Switching RT buffer (NEB), 1 µl of 75 μM T7-TSO (5′-/5Biosg/ACTCTAATACGACTCACTATAGGGAGAGGGCrGrGrG-3′), and 2 μl 10× Template Switching RT Enzyme Mix (NEB) were added to the mixture and the reaction was incubated at 42 °C for 90 min followed by 85 °C for 5 min. The second-strand synthesis was then performed by adding 100 μl Q5 Hot Start High Fidelity Master Mix (NEB), 10 μl RNase H (NEB) and 70 μl nuclease-free H_2_O to the cDNA mixture before incubation at 37 °C for 15 min, 95 °C for 1 min and 65 °C for 10 min. The double-stranded cDNA product was purified with 0.8× AMPure XP beads (Beckman Coulter). The IVT RNA control was prepared by T7 in vitro transcription using the purified cDNA product and HiScribe T7 High Yield RNA Synthesis Kit according to the manufacturer’s protocol. To remove cDNA template, IVT RNA was then treated with Turbo DNase and purified by Zymo-IC Column with RNA binding buffer.

#### BACS for Ψ detection

Around 50–200 ng ribo^−^ or poly-A^+^ RNA was fragmented by NEBNext Magnesium RNA Fragmentation Module at 94 °C for 4 min according to the manufacturer’s protocol and purified by Zymo-IC Column with RNA binding buffer. The fragmented RNA was mixed with 5 μl 10× T4 PNK reaction buffer (NEB), 5 μl T4 PNK (NEB) and 2.5 μl SUPERase•In RNase Inhibitor (Invitrogen) in a 50 µl final solution and incubated at 37 °C for 1 h. The 3′-repaired RNA was purified by Zymo-IC Column with RNA binding buffer and eluted with 10 μl nuclease-free H_2_O. The eluted RNA was then mixed with 1 μl synthetic 30mer spike-ins (2%) and 1 μl of 20 μM RNA adaptor (5′-/5rApp/AGATCGGAAGAGCGTCGTG/3SpC3/-3′), incubated at 70 °C for 2 min and immediately placed on ice. Next, 2.5 μl 10× T4 RNA Ligase reaction buffer (NEB), 1 μl SUPERase•In RNase Inhibitor, 7.5 μl 50% PEG 8000 (NEB) and 2 μl T4 RNA Ligase 2, truncated KQ (NEB) were added to the mixture and the reaction was incubated at 25 °C for 2 h followed by 16 °C for 14 h. To digest excess adaptors, the solution was further diluted to 47 μl with nuclease-free H_2_O and treated with 2 μl 5′-deadenylase (NEB) at 30 °C for 1 h followed by adding 1 μl RecJ_f_ (NEB) and incubation at 37 °C for 1 h. The 3′-ligated RNA was purified by Zymo-IC Column with RNA binding buffer and eluted with 12 μl nuclease-free H_2_O. A 10 μl aliquot was subjected to BACS library construction, while the remaining 2 μl was saved as control sample and diluted to 12.5 μl with nuclease-free H_2_O. For BACS, 1 M 2-bromoacrylamide (Enamine) was prepared by dissolving the solid in DMSO. Next, 10 μl 3′-ligated RNA was added into a 20 μl solution containing 250 mM 2-bromoacrylamide and 625 mM phosphate buffer (pH 8.5) and incubated at 85 °C for 30 min. The treated RNA was double purified by Micro Bio-Spin P-6 Tris Column (Bio-Rad) and Zymo-IC Column with RNA binding buffer and finally eluted with 12.5 μl nuclease-free H_2_O.

Both treated and control RNA samples were mixed with 1 μl of 2 μM RT primer (5′-ACACGACGCTCTTCCGATCT-3′) and 1 μl of 10 mM dNTP mix, incubated at 70 °C for 2 min and immediately placed on ice. Next, 4 μl 5× Maxima H^−^ RT buffer (Thermo), 0.5 μl RiboLock RNase Inhibitor (Thermo) and 1 μl Maxima H^−^ Reverse Transcriptase (Thermo) were added to the mixture and the reaction was incubated at 50 °C for 1 h. To digest excess RT primers, the solution was treated with 1 μl Exo I (NEB) and incubated at 37 °C for 30 min followed by adding 1 μl of 0.5 M EDTA (Sigma) to quench the reaction. To hydrolyze the RNA, 2.5 µl of 1 M sodium hydroxide (Sigma) was added and the solution was then incubated at 70 °C for 12 min followed by adding 2.5 µl of 1 M HCl (Sigma) to neutralize sodium hydroxide. The cDNA was finally purified with Dynabeads MyOne Silane (Invitrogen) and eluted with 13 µl nuclease-free H_2_O. The eluted cDNA was then mixed with 2 µl of 25 μM cDNA adaptor (5′-/5Phos/NNNNNNAGATCGGAAGAGCACACGTCTG/3SpC3/-3′), incubated at 70 °C for 2 min and immediately placed on ice. Next, 5 μl 10× T4 RNA Ligase reaction buffer, 25 μl 50% PEG 8000, 0.5 μl of 100 mM ATP (NEB), 3.5 μl DMSO (Thermo) and 1 μl T4 RNA Ligase 1, high concentration (NEB) were added to the mixture and the reaction was incubated at 25 °C for 16 h. The ligated cDNA was purified with Dynabeads MyOne Silane and eluted with 15 µl nuclease-free H_2_O. The eluted DNA was amplified with NEBNext Multiplex Oligos for Illumina (96 Unique Dual Index Primer Pairs) and NEBNext Ultra II Q5 Master Mix for 10–12 cycles according to the manufacturer’s protocol. The PCR products were purified with 0.8× AMPure XP beads and quantified with Qubit dsDNA HS Assay Kit (Thermo) according to the manufacturer’s protocol. BACS and control libraries were sequenced on a NextSeq 2000 (60-bp paired end reads) with no PhiX added.

#### Modified BID-seq

Library construction of modified BID-seq was conducted similarly with BACS except for the chemical conversion step. For BS treatment, revised BS and desulfonation reaction conditions of BID-seq were used^22^. Briefly, the 3′-ligated RNA was eluted in 10 μl nuclease-free H_2_O. An 8.5 μl aliquot was subjected to modified BID-seq library construction, while the remaining 1.5 μl was saved as a control sample. The 8.5 μl aliquot was mixed with 45 μl freshly prepared BS reagent (2.4 M Na_2_SO_3_ and 0.36 M NaHSO_3_, Sigma) and incubated at 70 °C for 3 h. The treated RNA was purified by Zymo-IC Column with RNA binding buffer. In-column desulfonation was performed using RNA desulfonation buffer (Zymo), with the column incubated at room temperature for 75 min.

#### Data preprocessing

Raw sequencing reads were processed by Cutadapt (v.4.2)^57^ to remove low-quality bases (--q 20) and short reads (--m 18), as well as to trim adaptors. 6mer unique molecular identifiers (UMIs) were extracted by UMI-tools extract (v.1.0.1)^58^ and used for deduplication. Paired reads were then merged into single reads using fastp (v.1.0.1)^59^.

#### Read alignment

Cleaned reads were first mapped to synthetic spike-ins and rRNA references using Bowtie 2 (v.2.4.4)^60^. The key parameters are as follows: bowtie2 -p 2 --no-unal --local -L 16 -N 1 --mp 4. Human rRNA sequences (NR_023363.1, NR_003285.3, NR_003286.4 and NR_003287.4) were downloaded from the National Center for Biotechnology Information (NCBI). The unaligned reads were subsequently mapped to snoRNA references and then to tRNA references, using the same parameters. Human snoRNA sequences that belong to the HUGO Gene Nomenclature Committee (HGNC) ‘small nucleolar RNAs’ gene group (https://www.genenames.org/) were downloaded from RefSeq (https://www.ncbi.nlm.nih.gov/refseq/). Duplicate snoRNA sequences were removed. High-confidence human tRNA sequences (hg38) were downloaded from GtRNAdb^61^. Only nonredundant tRNA sequences were kept and appended with a 3′-CCA end. Finally, unmapped reads were aligned to human genome (hg38) with GENCODE v.43 annotation by STAR (v.2.7.9a)^62^.

For RNA viruses, the following reference genomes were used: SARS-CoV-2 isolate Wuhan-Hu-1 (NCBI, NC_045512.2), recombinant HCV J6 (5′ UTR-NS2)/JFH1 (NCBI, JF343782.1), Zika virus isolate ZIKV/*H. sapiens*/Brazil/Natal/2015 (NCBI, NC_035889.1), HDV sequence from the pSVL(D3) plasmid^63^ (Addgene plasmid, 29335; https://www.addgene.org/29335/) and SINV (NCBI, NC_001547.1). For EBV samples, reads were aligned to the EBV genome, strain B95-8 (NCBI, V01555.2).

The aligned reads were then filtered and sorted using SAMtools (v.1.16.1)^64^. For synthetic spike-ins and rRNA, only reads with MAPQ ≥ 10 were kept. For snoRNA and tRNA, only reads with MAPQ ≥ 1 were kept. For mRNA, only uniquely mapped reads (-q 30) with a maximum of three mutation counts were kept. Deduplication was performed using UMI-tools dedup (v.1.0.1)^58^. Finally, mutations were counted by SAMtools mpileup (v.1.16.1)^64^ and cpup (v.0.1.0; https://github.com/y9c/cpup/). The sequencing metrics and sample information can be found in Supplementary Table 3.

#### Calling Ψ sites

BACS raw conversion rates were calculated as C/(T + C). The Ψ modification levels were calculated using the linear equation: Ψ modification level = (R–F)/(C–F), where R, F and C indicated raw conversion rates, motif-specific false-positive rates (from NNUNN spike-in for ncRNAs or IVT-BACS library for poly-A-tailed RNA) and motif-specific conversion rates (from NNΨNN spike-in), respectively. The following criteria were used to call Ψ sites in ncRNAs: (1) coverage ≥ 20 in both BACS and control libraries; (2) background conversion rate ≤ 0.01 or T-to-C mutation counts ≤ 2 in control libraries; (3) background T-to-R (R = A or G) mutation ratio ≤ 0.10 in control libraries; (4) Ψ modification level ≥ 0.05; (5) a *P* value was calculated for each site using the motif-specific false-positive rates and then adjusted following the Benjamini–Hochberg procedure; the adjusted *P* value is required to be <0.001; (6) consistently detected in all replicates. For calling cy-tRNA Ψ sites, criteria (4) and (6) were modified to require a Ψ modification level ≥ 0.10 in at least one of two replicates. Only Ψ sites identified in expressed cy-tRNA isodecoders were reported. The following criteria were used to call Ψ sites in poly-A-tailed RNA: (1) coverage ≥ 20 in BACS, control and IVT-BACS libraries; (2) background conversion rate ≤ 0.01 or T-to-C mutation counts ≤ 2 in control libraries; conversion rate in BACS libraries higher than that in IVT-BACS libraries; (3) background T-to-R (R = A or G) mutation ratio ≤ 0.10 in control libraries; (4) Ψ modification level ≥ 0.05; (5) a contingency table test was performed for each site between BACS and IVT-BACS libraries; the *P* value is required to be <0.01. Statistical analyses were performed in R (v.4.0.3). The full list of Ψ sites identified in the HeLa transcriptome can be found in Supplementary Tables 4–9.

#### Calling A-to-I editing sites

A-to-I editing sites were called based on the ICE-seq protocol^65^, with minor modifications. Candidate A-to-I editing sites are required to meet the following criteria: (1) coverage ≥ 10 in control libraries; (2) A-to-G mutation ratio ≥ 0.05 in control libraries; (3) A-to-Y (Y = C or T) mutation ratio < 0.05 in control libraries. Supporting reads for the candidate sites are required to meet the following criteria: (1) supporting reads containing an indel call within 5 bp upstream or downstream to the candidate sites were filtered out; (2) supporting reads containing mismatches other than the A-to-G mutation were filtered out; (3) total counts of supporting reads ≥ 3. A ΔG score for each candidate site was calculated as follows:

$$\Delta {\rm{G}}={\log }_{2}\left(\frac{{{\rm{N}}}_{{\rm{G}}}\left({\rm{BACS}}\right)+1}{{{\rm{N}}}_{{\rm{G}}}\left({\rm{control}}\right)+1}\cdot \frac{{\rm{N}}\left({\rm{control}}\right)}{{\rm{N}}\left({\rm{BACS}}\right)}\right)$$

where N_G_ and N denote the counts of A-to-G mutation and total mapped bases, respectively. The ΔG score is required to be ≤−1. Finally, candidate A-to-I editing sites registered as common SNPs in the dbSNP^66^ were removed. The full list of A-to-I editing sites identified in the HeLa transcriptome can be found in Supplementary Table 10.

#### RNA structure visualization

The RNA–RNA interactions were visualized using r2r (v.1.0.6)^67^. The snoRNA–rRNA interactions were adapted from snoRNA Atlas^21^.

#### Downstream analysis

The snoRNA box and guide sequences were downloaded from snoDB 2.0 (ref. ^68^). In the metagene analysis, snoRNA sequences that displayed considerable similarity were streamlined, retaining only one representative snoRNA. The annotation of Ψ sites identified in poly-A-tailed RNA was performed using bedtools intersect (v.2.30.0)^69^ with GENCODE v.43 annotation. Read counts obtained from featureCounts (v.1.6.4)^70^ were normalized based on sequencing depth and gene length using the transcripts per million method. GO analysis was performed with mRNA Ψ sites using enrichR (v.3.2)^71^. Sequence logos were generated using ggseqlogo (v.0.1)^72^ in R (v.4.3.1).

#### Calling PUS-dependent Ψ sites

The following criteria were used to call PUS-dependent Ψ sites: (1) coverage ≥ 20 in both WT and KO samples; (2) Ψ modification level ≥ 0.05 in WT samples; (3) T-to-C mutation counts ≥ 10 in WT samples; (4) Ψ modification level ≤ 0.01 in KO samples. For downregulated Ψ sites, we required the reduction in Ψ modification level to be ≥0.20.

#### Published data

Related published data were downloaded from the Gene Expression Omnibus (GEO) database: BID-seq for HeLa cells (GSE179798)^12^. BID-seq data were processed using the original pipeline (BID-seq^12^) and updated pipeline (BID-pipe^22^), respectively.

### Reporting summary

Further information on research design is available in the Nature Portfolio Reporting Summary linked to this article.

## Online content

Any methods, additional references, Nature Portfolio reporting summaries, source data, extended data, supplementary information, acknowledgements, peer review information; details of author contributions and competing interests; and statements of data and code availability are available at 10.1038/s41592-024-02439-8.

## Supplementary information

Supplementary Information Supplementary Figs. 1–17 and Supplementary Tables 1 and 2.

Reporting Summary

Supplementary Table 3–13

## Source data

Source Data Fig. 1 Statistical source data.

Source Data Fig. 2 Statistical source data.

Source Data Fig. 3 Statistical source data.

Source Data Fig. 4 Statistical source data.

Source Data Fig. 5 Statistical source data.

Source Data Fig. 6 Statistical source data.

## Data Availability

All sequencing data are available on the GEO database under accession GSE241849. Published data were downloaded from the GEO database: BID-seq for HeLa cells (GSE179798)^12^. All relevant additional data have been published with the manuscript, either as part of the main text or in Supplementary Information. Source data are provided with this paper.

## Code availability

The analysis scripts are available at https://github.com/lkong888/bacs/.
