# Supplementary material for: Absolute quantitative and base-resolution sequencing reveals comprehensive landscape of pseudouridine across the human transcriptome
Source: Nat Methods. 2024 Sep 30;21(11):2024–33. doi: 10.1038/s41592-024-02439-8 (PMC11541003; doi:10.1038/s41592-024-02439-8)
Supplement: Supplementary file 1 — Supplementary Figs. 1–17 and Supplementary Tables 1 and 2. [file 41592_2024_2439_MOESM1_ESM.pdf]

nature methods

Supplementary information https://doi.org/10.1038/s41592-024-02439-8

Absolute quantitative and base-resolution sequencing reveals comprehensive landscape of pseudouridine across the human transcriptome

In the format provided by the authors and unedited

Table of Contents

**Supplementary Fig. 1**	Performance of BACS on model RNA
**Supplementary Fig. 2**	BACS validated known Ψ sites in human rRNA
**Supplementary Fig. 3**	Comparison of BACS and BS-based methods for Ψ detection in human cy-rRNAs
**Supplementary Fig. 4**	BACS identified conserved and novel Ψ sites in human spliceosomal snRNA
**Supplementary Fig. 5**	BACS detected abundant Ψ sites in human snoRNAs
**Supplementary Fig. 6**	Potential involvement of Ψ in regulating the guiding activity of human snoRNAs
**Supplementary Fig. 7**	BACS revealed a comprehensive map of Ψ in human tRNA
**Supplementary Fig. 8**	Comparison of BACS and PRAISE for Ψ detection in human mt-tRNAs
**Supplementary Fig. 9**	Analysis of BACS libraries for polyA-tailed RNA
**Supplementary Fig. 10**	Sequence context and codon preference of Ψ in HeLa mRNA
**Supplementary Fig. 11**	Comparison of mRNA Ψ sites identified by BACS with published datasets
**Supplementary Fig. 12**	BACS enabled simultaneous detection of m^1A with Ψ
**Supplementary Fig. 13**	BACS assigned responsible PUS enzymes for Ψ sites in the HeLa transcriptome
**Supplementary Fig. 14**	Absence of Ψ in transcripts and genomes of RNA viruses
**Supplementary Fig. 15**	Validation of Ψ_{114} site in EBER2
**Supplementary Fig. 16**	Validation of TRUB1-KO, PUS7-KO, and PUS1-KO HeLa cell lines
**Supplementary Fig. 17**	Unprocessed western blots related to Supplementary Fig. 16
**Supplementary Table 1**	RNA oligonucleotide sequences in this work
**Supplementary Table 2**	Compound-dependent UHPLC-MS/MS parameters used for nucleoside quantification

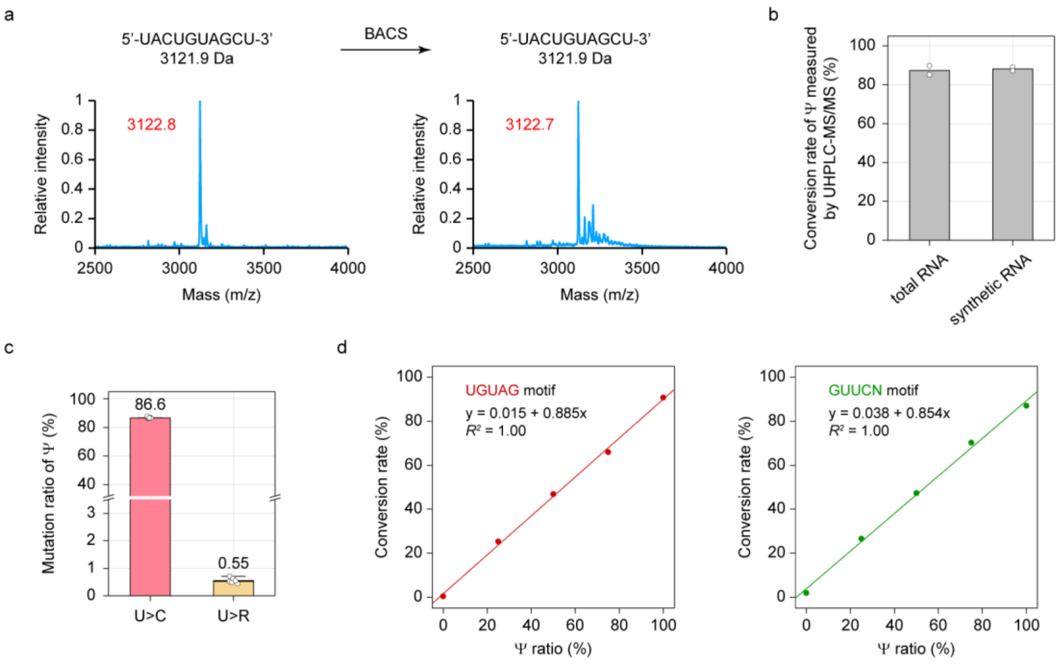

Supplementary Fig. 1 | Performance of BACS on model RNA. (a) MALDI characterization of BACS labeling of a 10mer unmodified RNA oligonucleotide. Calculated mass is shown in black. Observed mass is shown in red. Data are representative of two independent experiments. **(b)** Conversion rates of Ψ in HeLa total RNA and 1.8-kb 10% Ψ-modified synthetic RNA upon BACS treatment, quantified by UHPLC-MS/MS. Data are presented as means of two independent experiments. **(c)** Mutation ratios of Ψ sites in 72mer model RNA after BACS treatment. Data are shown as means ± s.d. of six independent experiments (n = 6). **(d)** BACS calibration curve for quantification of Ψ stoichiometry in UGUAG (red) and GUUCN (green) motif. Data are representative of two independent experiments.

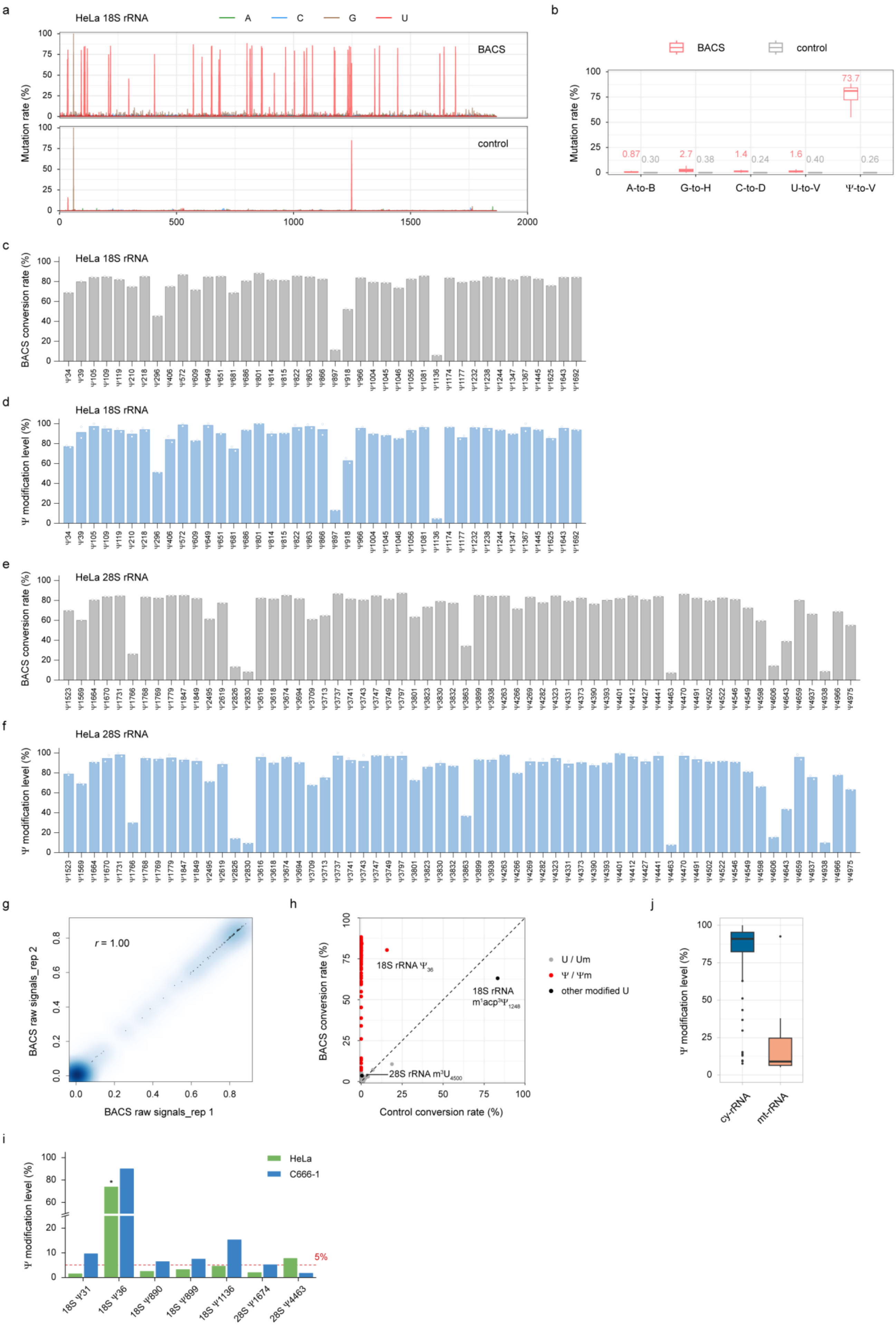
a
HeLa 18S rRNA
A
C
G
U
BACS
control
Mutation rate (%)
b
BACS
control
Mutation rate (%)
0.87
0.30
2.7
0.38
1.4
0.24
1.6
0.40
73.7
0.26
A-to-B
G-to-H
C-to-D
U-to-V
Ψ-to-V
c
HeLa 18S rRNA
BACS conversion rate (%)
Ψ34 Ψ39 Ψ105 Ψ109 Ψ119 Ψ210 Ψ218 Ψ296 Ψ406 Ψ572 Ψ609 Ψ649 Ψ651 Ψ681 Ψ686 Ψ801 Ψ814 Ψ815 Ψ822 Ψ863 Ψ866 Ψ897 Ψ918 Ψ966 Ψ1004 Ψ1045 Ψ1046 Ψ1056 Ψ1081 Ψ1136 Ψ1174 Ψ1177 Ψ1232 Ψ1238 Ψ1244 Ψ1347 Ψ1367 Ψ1445 Ψ1625 Ψ1643 Ψ1692
d
HeLa 18S rRNA
Ψ modification level (%)
e
HeLa 28S rRNA
BACS conversion rate (%)
Ψ1523 Ψ1569 Ψ1664 Ψ1670 Ψ1731 Ψ1766 Ψ1768 Ψ1769 Ψ1779 Ψ1847 Ψ1849 Ψ2495 Ψ2619 Ψ2826 Ψ2830 Ψ3616 Ψ3618 Ψ3674 Ψ3694 Ψ3709 Ψ3713 Ψ3737 Ψ3741 Ψ3743 Ψ3747 Ψ3749 Ψ3797 Ψ3801 Ψ3823 Ψ3830 Ψ3832 Ψ3863 Ψ3899 Ψ3938 Ψ4263 Ψ4266 Ψ4269 Ψ4282 Ψ4323 Ψ4331 Ψ4373 Ψ4390 Ψ4393 Ψ4401 Ψ4412 Ψ4427 Ψ4441 Ψ4463 Ψ4470 Ψ4491 Ψ4502 Ψ4522 Ψ4546 Ψ4549 Ψ4598 Ψ4606 Ψ4643 Ψ4659 Ψ4937 Ψ4938 Ψ4966 Ψ4975
f
HeLa 28S rRNA
Ψ modification level (%)
g
r = 1.00
BACS raw signals_rep 2
BACS raw signals_rep 1
h
18S rRNA Ψ36
18S rRNA m1acp3Ψ1248
28S rRNA m3U4500
BACS conversion rate (%)
Control conversion rate (%)
U / Um
Ψ / Ψm
other modified U
j
Ψ modification level (%)
cy-rRNA
mt-rRNA
i
Ψ modification level (%)
HeLa
C666-1
5%
18S Ψ31
18S Ψ36
18S Ψ890
18S Ψ899
18S Ψ1136
28S Ψ1674
28S Ψ4463

Supplementary Fig. 2 | BACS validated known Ψ sites in human rRNA. (a) BACS and control mutation rates of all bases in HeLa 18S rRNA. **(b)** Mutation rates of Ψ and other bases in HeLa cy-rRNAs. For boxplots, boxes represent the 25th to 75th percentiles with a line at the median; whiskers correspond to 1.5 times the interquartile range; mean values are listed above each plot (A-to-B, *n* = 1268; G-to-H, *n* = 2459; C-to-D, *n* = 2272; U-to-V, *n* = 1113; Ψ-to-V, *n* = 105). B = C, G or U; H = A, C or U; D = A, G or U; V = A, C or G. **(c)** BACS conversion rates of Ψ sites identified in HeLa 18S rRNA. Data are presented as means of two independent experiments. **(d)** Modification levels of Ψ sites detected in HeLa 18S rRNA. Data are presented as means of two independent experiments. Ψ modification levels were calculated using the calibration curves. **(e)** BACS conversion rates of Ψ sites identified in HeLa 28S rRNA. Data are presented as means of two independent experiments. **(f)** Modification levels of Ψ sites detected in HeLa 28S rRNA. Data are presented as means of two independent experiments. Ψ modification levels were calculated using the calibration curves. **(g)** Correlation density plot between two biological replicates of BACS. The color scale represents density. **(h)** Comparison of the conversion rates in HeLa cy-rRNAs between BACS and control samples. **(i)** Comparison of the modification levels of selected Ψ sites in cy-rRNAs between HeLa (green) and C666-1 (blue) cell lines. * Ψ_{36} was found to overlap with a SNP site in HeLa cells. Consequently, its modification level was inferred by comparing the mutation rates between BACS and control libraries. **(j)** Comparison of the modification levels of Ψ sites in HeLa cy-rRNAs and mt-rRNAs. For boxplots, boxes represent the 25th to 75th percentiles with a line at the median; whiskers correspond to 1.5 times the interquartile range; dots indicate outliers (cy-rRNA, *n* = 104; mt-rRNA, *n* = 9).

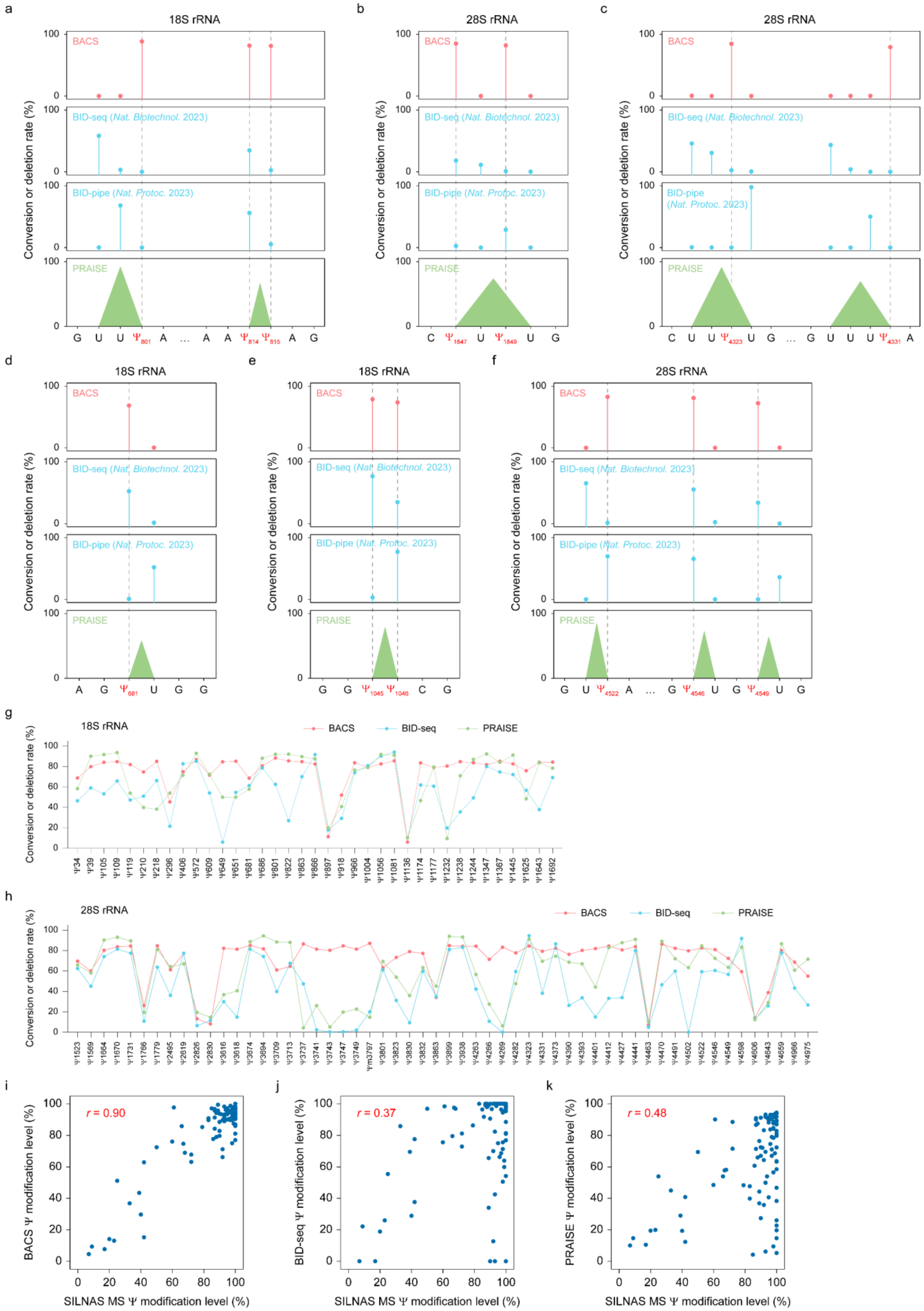
a
18S rRNA
b
28S rRNA
c
28S rRNA
d
18S rRNA
e
18S rRNA
f
28S rRNA
BACS
BID-seq (Nat. Biotechnol. 2023)
BID-pipe (Nat. Protoc. 2023)
PRAISE
Conversion or deletion rate (%)
g
18S rRNA
h
28S rRNA
BACS
BID-seq
PRAISE
i
r = 0.90
BACS Ψ modification level (%)
j
r = 0.37
BID-seq Ψ modification level (%)
k
r = 0.48
PRAISE Ψ modification level (%)
SILNAS MS Ψ modification level (%)

Supplementary Fig. 3 | Comparison of BACS and BS-based methods for Ψ detection in human cy-rRNAs. (a–f) Examples of BACS, BID-seq, BID-pipe, and PRAISE results in selected consecutive uridine regions of 18S and 28S rRNA. Due to their deletion signals, BS-based methods cannot determine the exact position or identify the number of Ψ within consecutive uridine contexts. By default, the aligner will position the deletion at the 5'-most uridine, as shown in the BID-seq panel. After realignment, this issue cannot be fully resolved, as shown in the BID-pipe panel. PRAISE considers consecutive uridines as a whole for Ψ calling, resulting in broad peak signals. **(g,h)** Comparison of the conversion rates of BACS (pink) with the deletion rates of BID-seq (blue) and PRAISE (green) for selected Ψ sites in 18S rRNA **(g)** and 28S rRNA **(h)**. Because BID-seq and PRAISE cannot quantify multiple Ψ sites (≥ 2) located in the same consecutive uridine context, these sites were excluded. **(i–k)** Correlation of cy-rRNA Ψ modification levels reported by SILNAS MS with those reported by BACS **(i)**, BID-seq **(j)**, and PRAISE **(k)**.

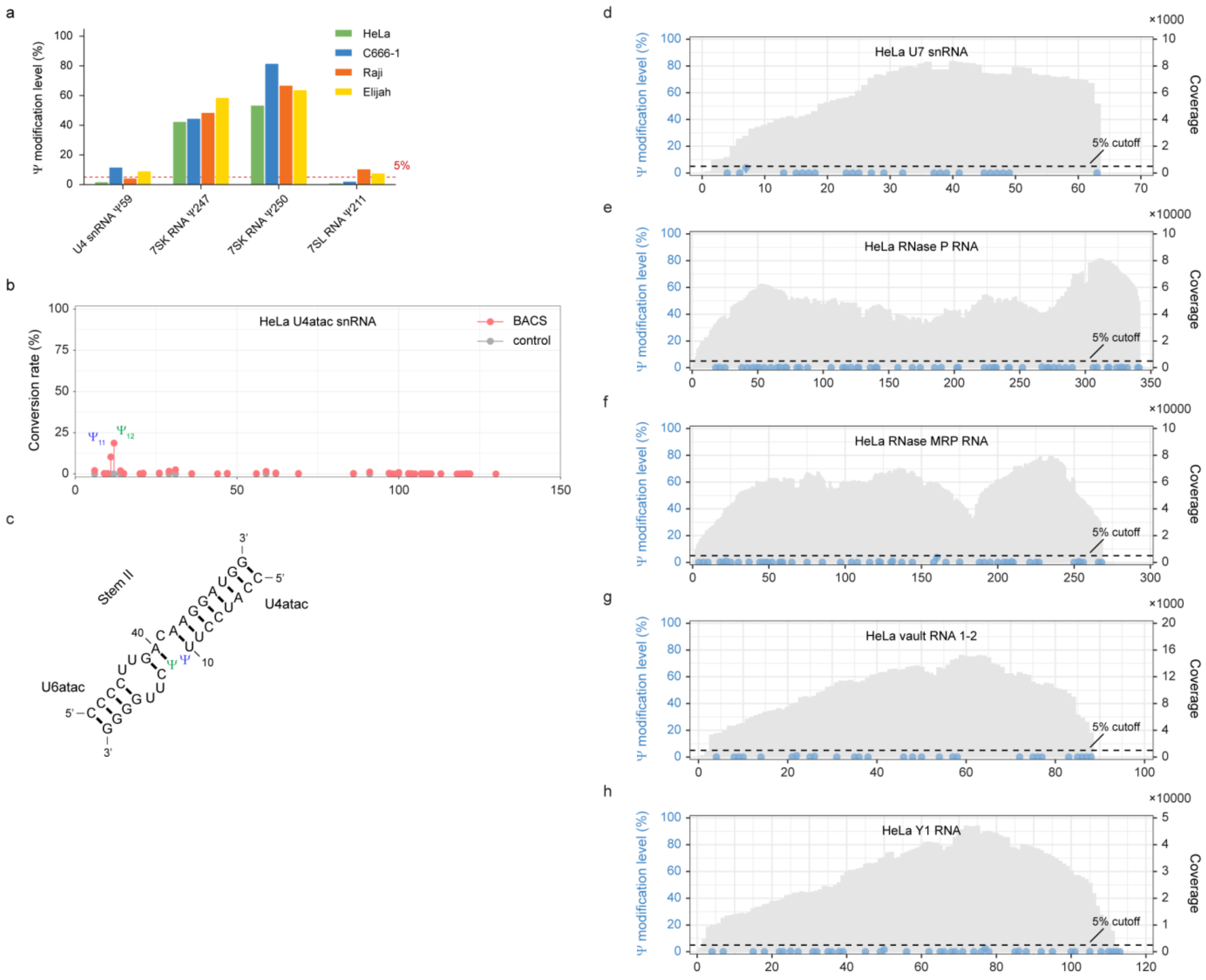

Supplementary Fig. 4 | BACS identified conserved and novel Ψ sites in human spliceosomal snRNA. (a) Comparison of the modification levels of selected Ψ sites in U4 snRNA, 7SK RNA, and 7SL RNA across HeLa (green), C666-1 (blue), Raji (orange), and Elijah (yellow) cell lines. **(b)** Conversion rates of BACS (pink) and control (grey) samples in U4atac snRNA, showing the novel (blue) and known (green) Ψ site. Data are presented as means of two independent experiments. **(c)** Base pairing interactions between U4atac and U6atac snRNAs in stem II region. Blue and green color denote the novel Ψ_{11} and known Ψ_{12} site, respectively. **(d–h)** Ψ modification levels in U7 snRNA **(d)**, RNase P RNA **(e)**, RNase MRP RNA **(f)**, vault RNA **(g)**, and Y RNA **(h)**. The sequencing coverage of each RNA was plotted in grey. Only one example was shown for vault RNA and Y RNA, respectively.

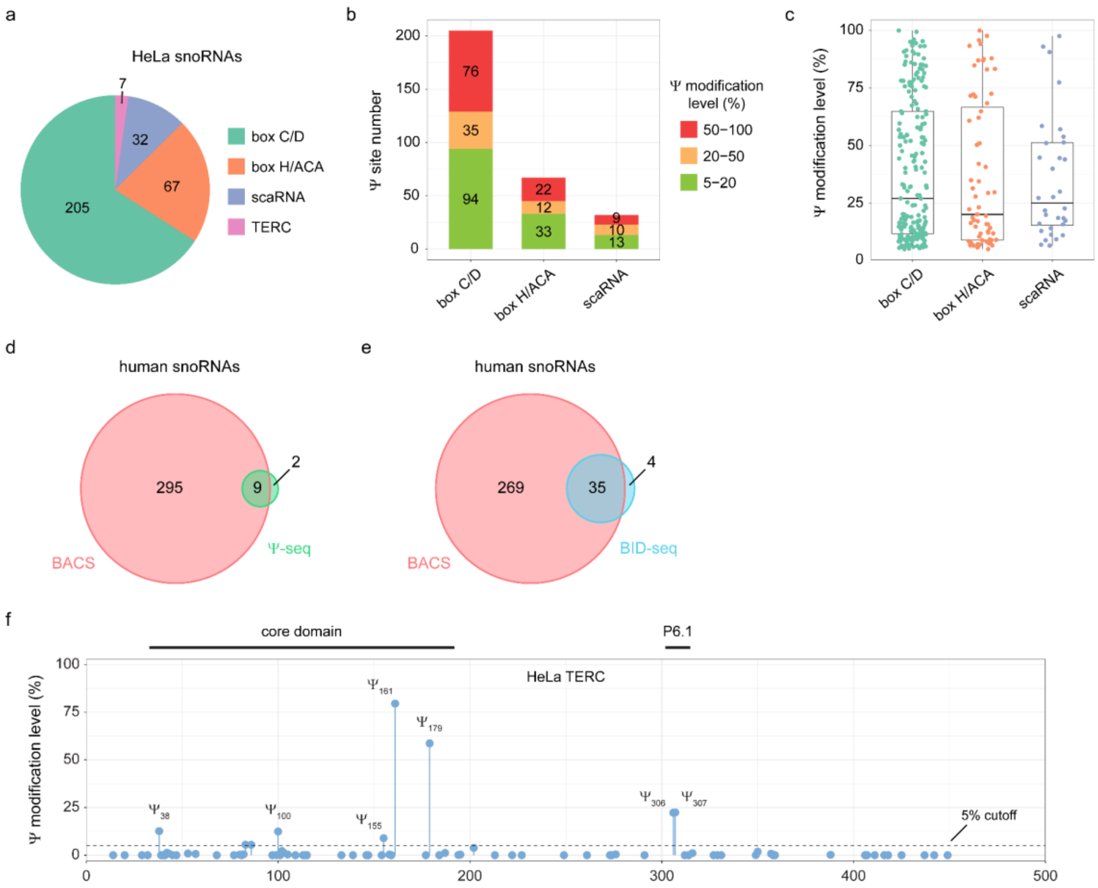

Supplementary Fig. 5 | BACS detected abundant Ψ sites in human snoRNAs. (a) Numbers of Ψ sites identified in HeLa snoRNAs and TERC. **(b)** Numbers of Ψ sites with high (50–100%, red), medium (20–50%, yellow), and low (5–20%, green) modification levels identified in HeLa box C/D snoRNAs, box H/ACA snoRNAs, and scaRNAs. **(c)** Modification level distributions of Ψ sites in HeLa box C/D snoRNAs, box H/ACA snoRNAs, and scaRNAs. Boxplots visualize all Ψ sites in each class of snoRNAs; boxes represent the 25th to 75th percentiles with a line at the median; whiskers correspond to 1.5 times the interquartile range (box C/D, n = 205; box H/ACA, n = 67; scaRNA, n = 32). **(d)** Venn diagram illustrating the overlap of Ψ sites detected in human snoRNAs between BACS and Ψ-seq. **(e)** Venn diagram illustrating the overlap of Ψ sites detected in human snoRNAs between BACS and BID-seq. **(f)** Ψ modification levels in HeLa TERC, with each identified Ψ site labeled accordingly. Data are presented as means of two independent experiments.

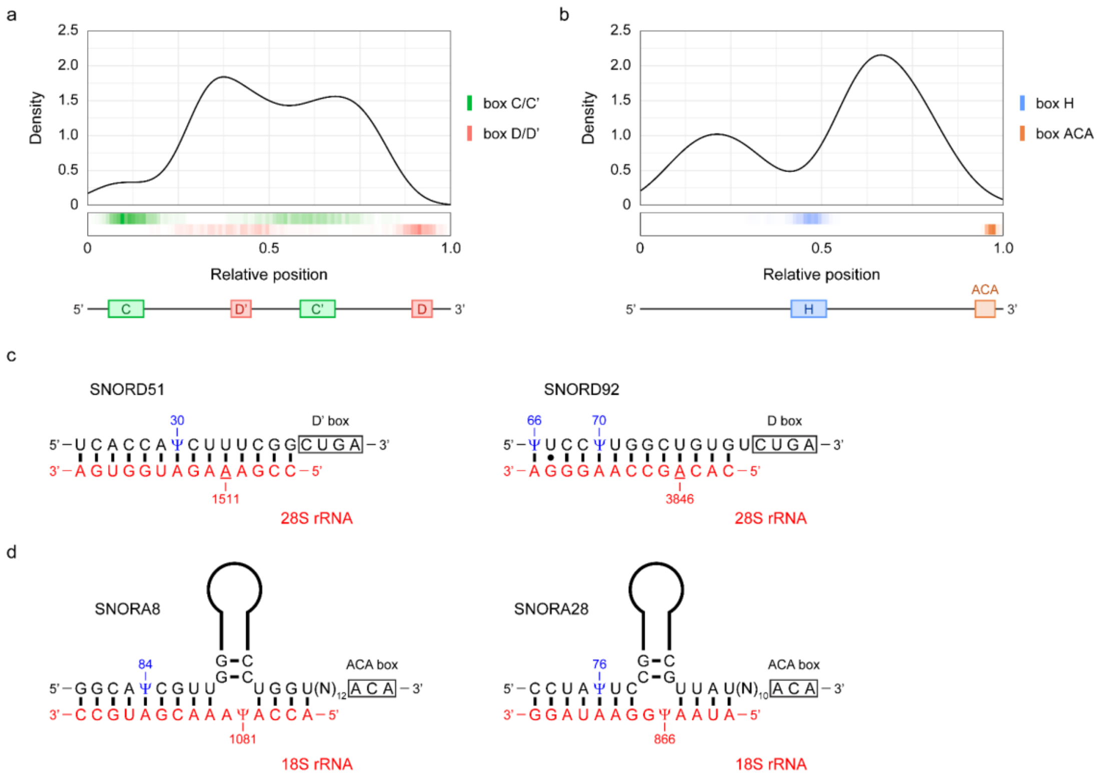

Supplementary Fig. 6 | Potential involvement of Ψ in regulating the guiding activity of human snoRNAs. (a) Metagene profile of Ψ sites in box C/D snoRNAs. Red and green color denote the box C/C' and D/D', respectively. **(b)** Metagene profile of Ψ sites in box H/ACA snoRNAs. Orange and blue color denote the box H and ACA, respectively. **(c,d)** Potential base pairing interactions between snoRNAs (black) and their targets in rRNA (red): **c.** box C/D snoRNAs and **d.** box H/ACA snoRNAs. Identified snoRNA Ψ sites are highlighted in blue. 2'-*O*-methylation targets in rRNA are underlined. Structures are adapted from snoRNA Atlas[1].

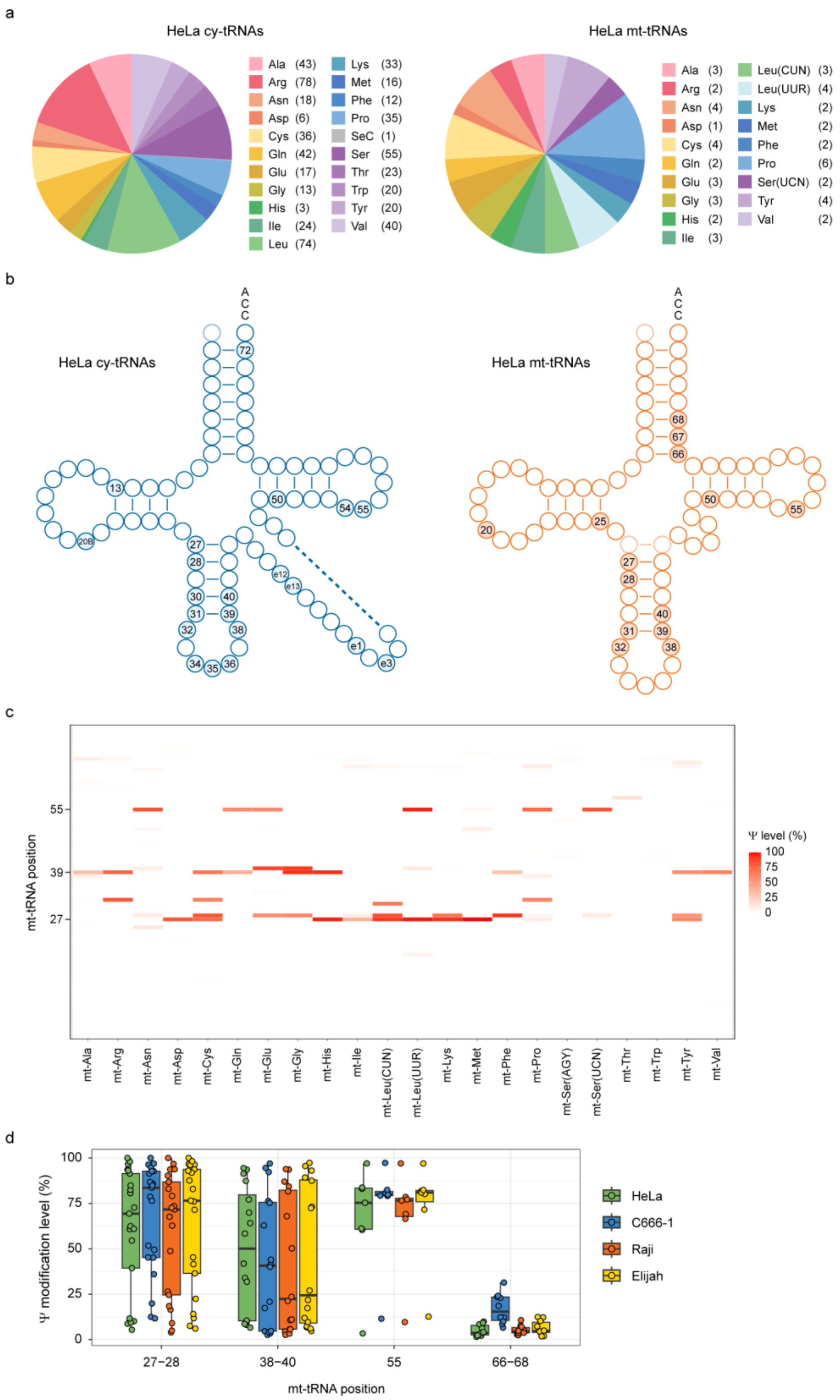

Supplementary Fig. 7 | BACS revealed a comprehensive map of Ψ in human tRNA. (a) Distributions of Ψ sites identified in each cy-tRNA (left) and mt-tRNA (right) isotype from HeLa cells. **(b)** Integrated view of the Ψ profiles of HeLa cy-tRNAs (left) and mt-tRNAs (right). **(c)** Heatmap of the Ψ modification levels in HeLa mt-tRNAs. **(d)** Comparison of the modification levels of Ψ sites at selected positions of mt-tRNAs across HeLa (green), C666-1 (blue), Raji (orange), and Elijah (yellow) cell lines. Boxplots visualize all Ψ sites at each position; boxes represent the 25th to 75th percentiles with a line at the median; whiskers correspond to 1.5 times the interquartile range (tRNA position: 27–28, n = 21; 38–40, n = 16; 55, n = 7; 66–68, n = 10).

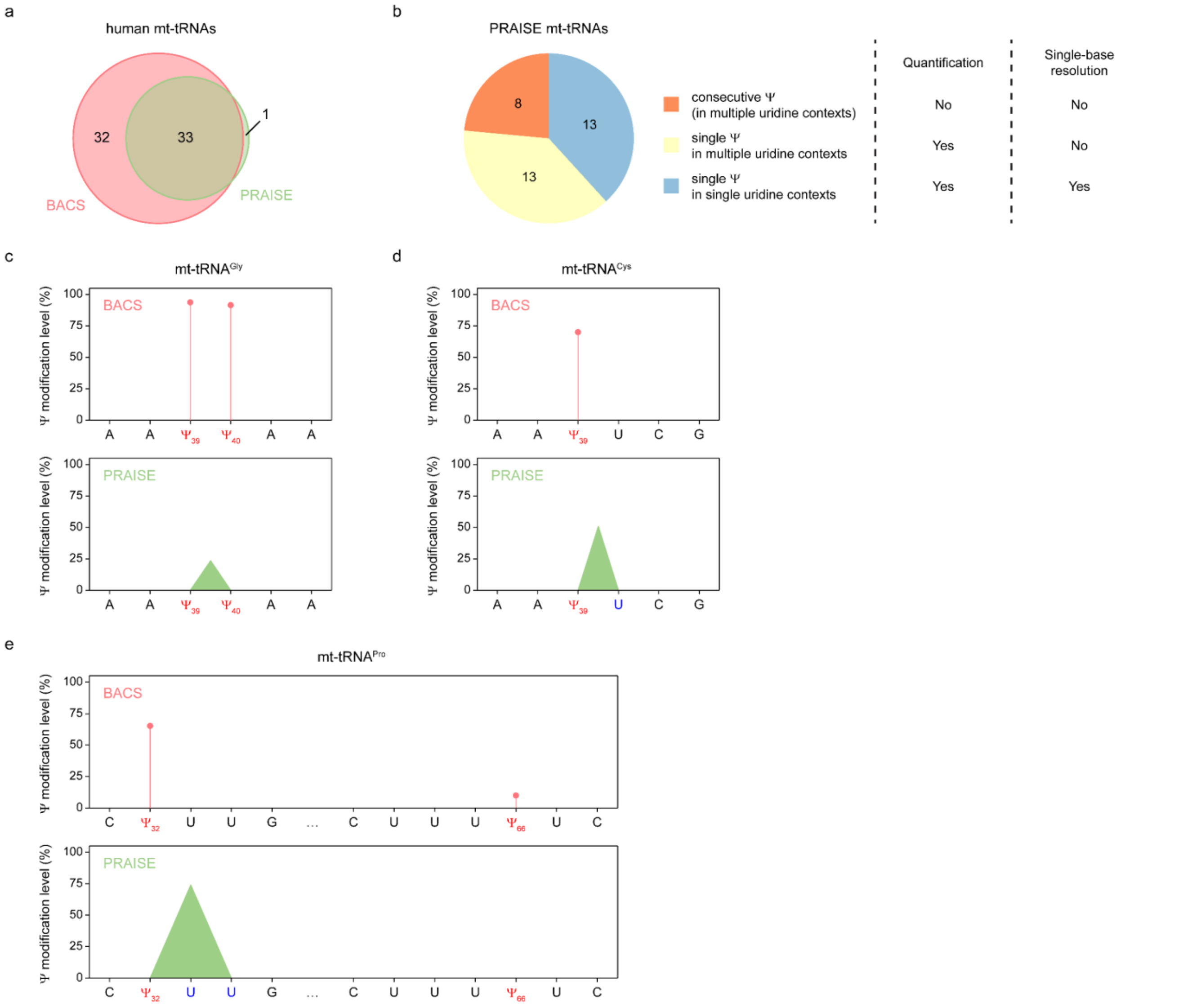

Supplementary Fig. 8 | Comparison of BACS and PRAISE for Ψ detection in human mt-tRNAs. (a) Venn diagram illustrating the overlap of Ψ sites detected in human mt-tRNAs between BACS and PRAISE. **(b)** Distribution of mt-tRNA Ψ sites identified by PRAISE. Only single Ψ site in single uridine contexts can be quantitatively identified by PRAISE at single-base resolution. **(c–e)** Examples of BACS and PRAISE results in selected consecutive uridine regions of human mt-tRNAs. Due to their deletion signals, BS-based methods cannot determine the exact position or identify the number of Ψ within consecutive uridine contexts. PRAISE considers consecutive uridines as a whole for Ψ calling, resulting in broad peak signals.

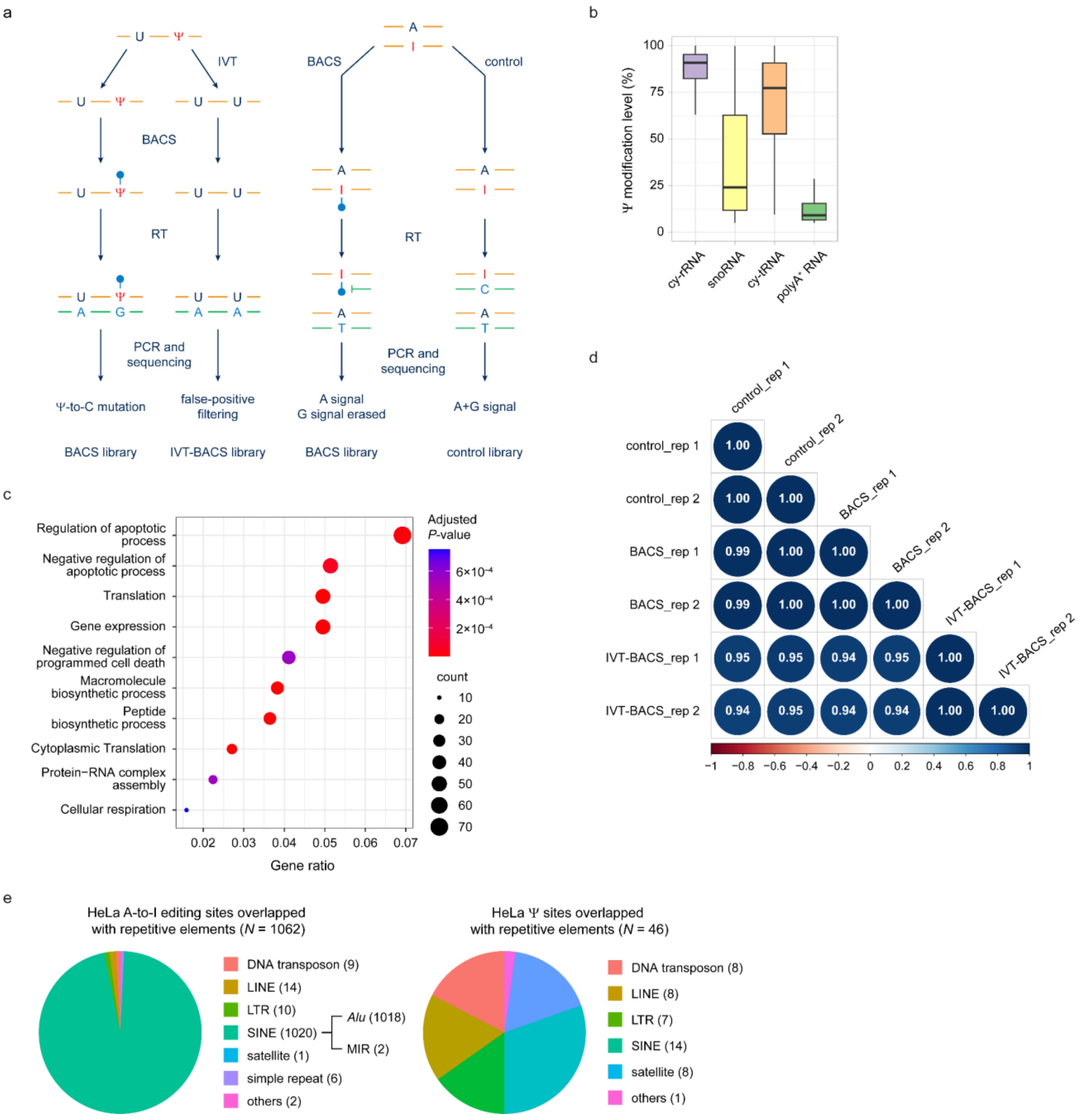

Supplementary Fig. 9 | Analysis of BACS libraries for polyA-tailed RNA. (a) Schematic overview of Ψ and A-to-I editing site identification in polyA-tailed RNA. Ψ site calling is based on BACS and IVT-BACS libraries. A-to-I editing site calling is based on BACS and control libraries. **(b)** Comparison of the Ψ modification levels in different RNA species. For boxplots, boxes represent the 25th to 75th percentiles with a line at the median; whiskers correspond to 1.5 times the interquartile range (cy-rRNA, n = 104; snoRNA, n = 304; cy-tRNA, n = 609; polyA-tailed RNA, n = 1335). **(c)** Gene ontology enrichment analysis (biological process) for HeLa mRNA Ψ sites. **(d)** Correlation of HeLa RNA expression levels between BACS, control, and IVT-BACS libraries. Pearson's r values are shown. **(e)** Distribution of HeLa A-to-I editing and Ψ sites overlapped with repetitive elements.

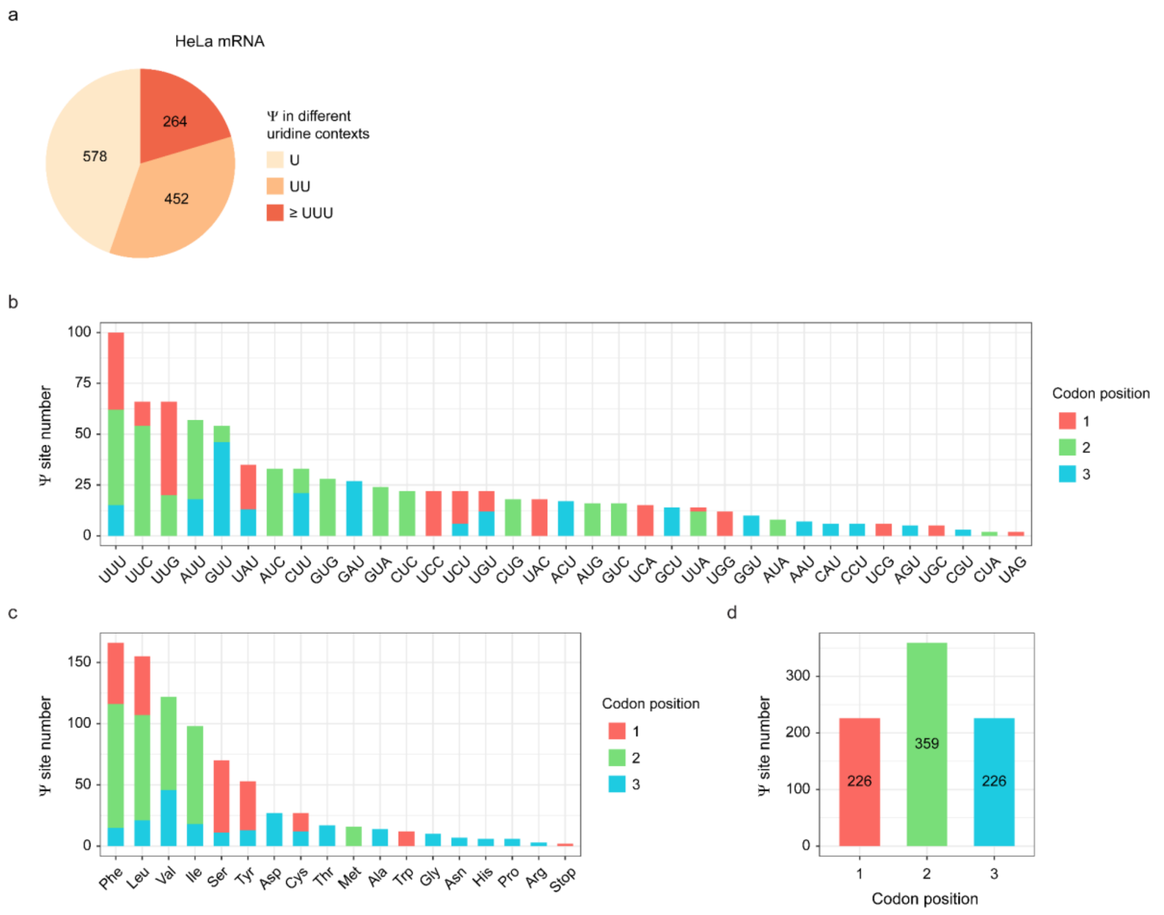

Supplementary Fig. 10 | Sequence context and codon preference of Ψ in HeLa mRNA. (a) Distribution of mRNA Ψ sites within single and consecutive uridine contexts. **(b,c)** Numbers of mRNA Ψ sites located in different codons **(b)** and codons encoding different amino acids **(c)**. Red, green, and blue color denote the first, second, and third codon position, respectively. **(d)** Numbers of mRNA Ψ sites located in different codon positions.

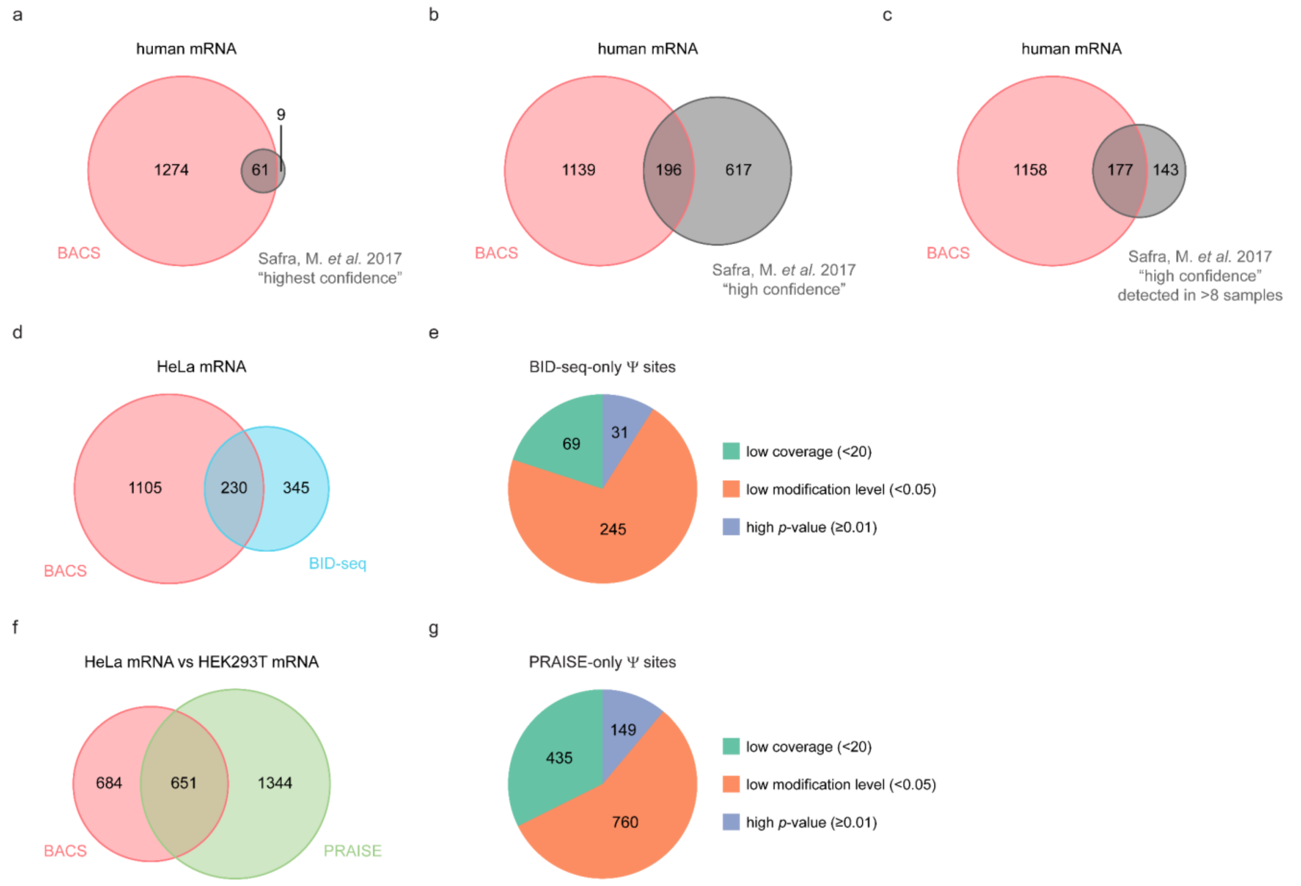

Supplementary Fig. 11 | Comparison of mRNA Ψ sites identified by BACS with published datasets. (a) Venn diagram illustrating the overlap of mRNA Ψ sites between BACS and the "highest confidence" list in a consolidated CMC-based dataset[2]. **(b)** Venn diagram illustrating the overlap of mRNA Ψ sites between BACS and the "high confidence" list in a consolidated CMC-based dataset[2]. **(c)** Venn diagram illustrating the overlap of mRNA Ψ sites between BACS and the "high confidence" list in a consolidated CMC-based dataset[2]. Only Ψ sites consistently detected across more than 8 samples in the "high confidence" list were considered. **(d)** Venn diagram illustrating the overlap of mRNA Ψ sites between BACS and BID-seq. **(e)** Distribution of BID-seq-only Ψ sites in BACS dataset. **(f)** Venn diagram illustrating the overlap of mRNA Ψ sites between BACS and PRAISE. **(g)** Distribution of PRAISE-only Ψ sites in BACS dataset.

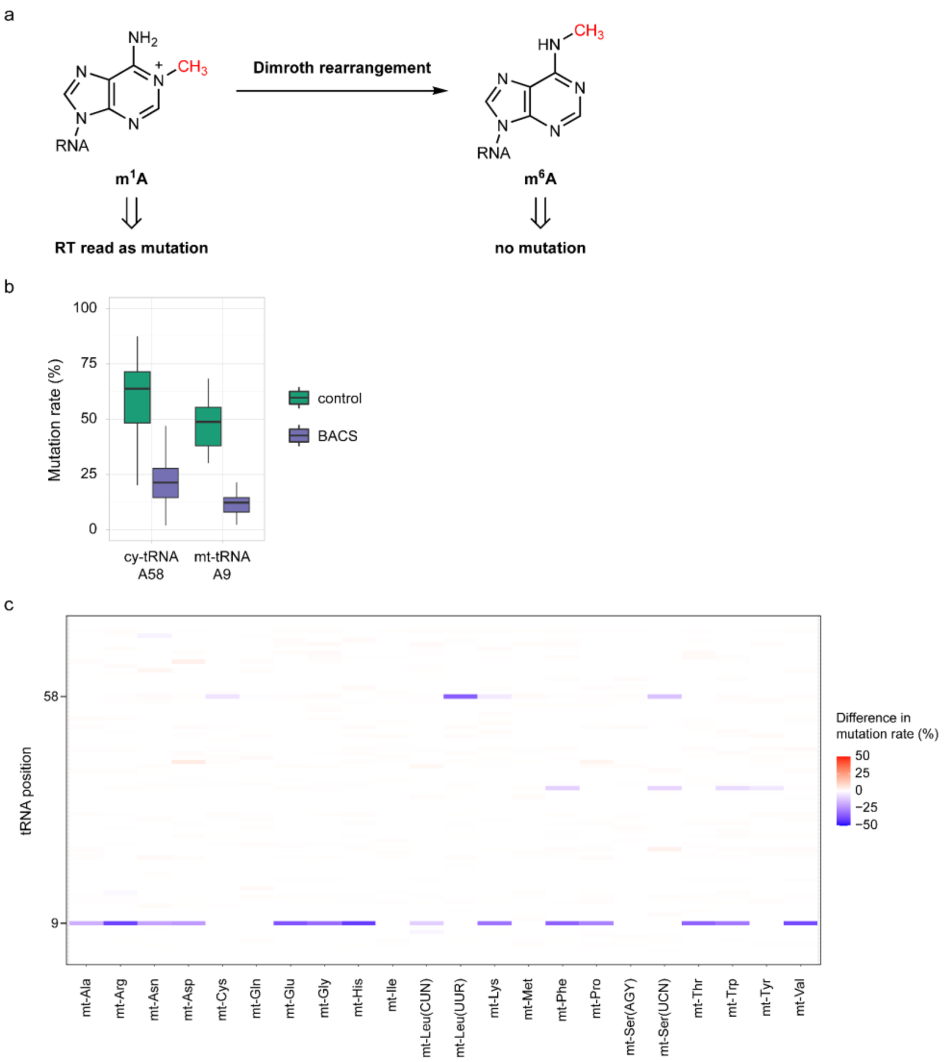

Supplementary Fig. 12 | BACS enabled simultaneous detection of m^{1}A with Ψ. (a) Schematic overview of Dimroth rearrangement of m^{1}A to m^{6}A. **(b)** Comparison of the mutation rates of known m^{1}A sites in control (green) and BACS (purple) samples. For boxplots, boxes represent the 25th to 75th percentiles with a line at the median; whiskers correspond to 1.5 times the interquartile range (cy-tRNA A58, n = 170; mt-tRNA A9, n = 14). **(c)** Heatmap showing the changes in mutation rates of all adenosine sites in human mt-tRNAs upon BACS treatment. Red and blue color indicate an increase and decrease of mutation rates, respectively.

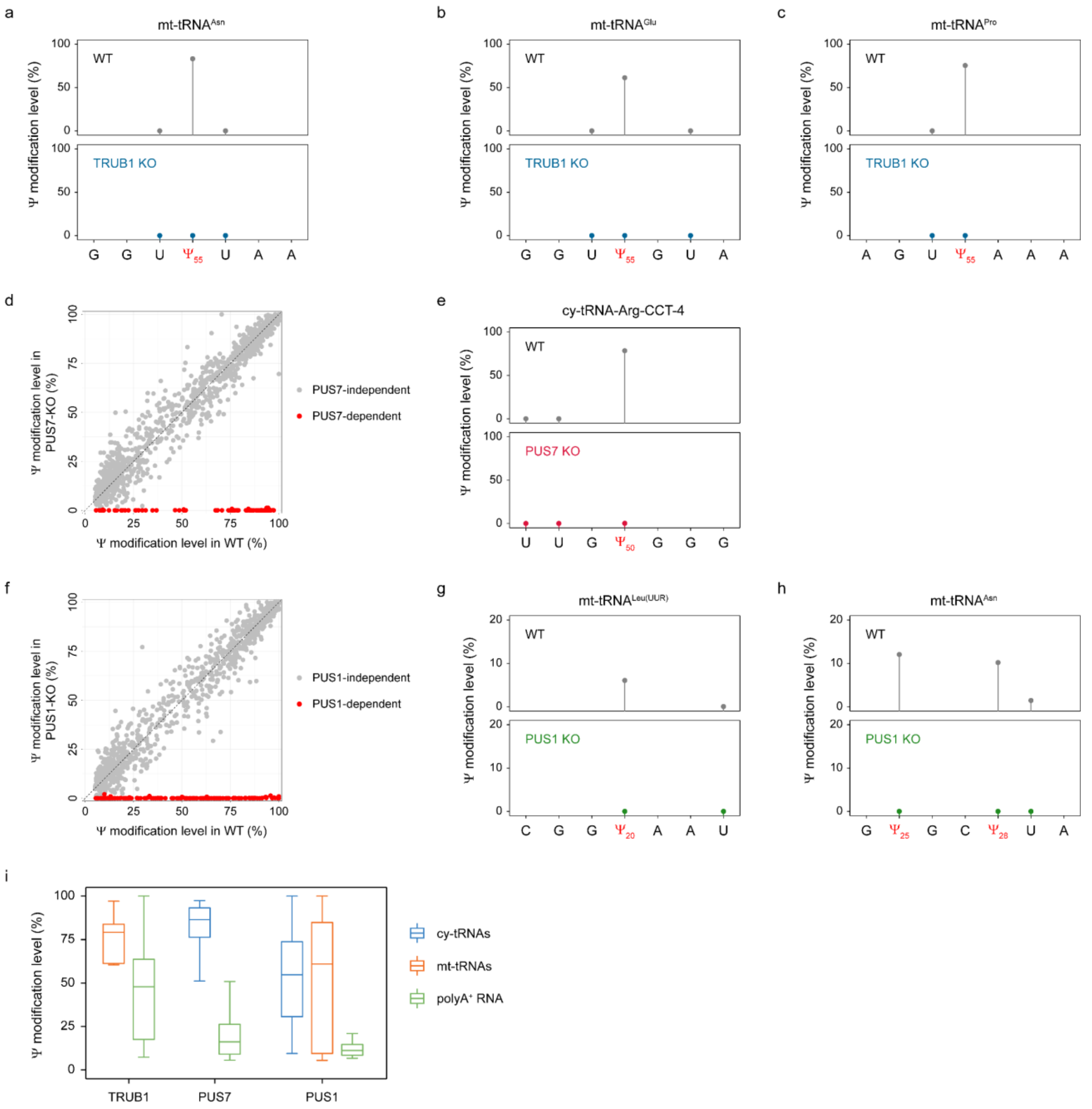

Supplementary Fig. 13 | BACS assigned responsible PUS enzymes for Ψ sites in the HeLa transcriptome. (a–c) Comparison of the modification levels of Ψ55 in mt-tRNA^{Asn} **(a)**, mt-tRNA^{Glu} **(b)**, and mt-tRNA^{Pro} **(c)** between WT and TRUB1-KO cell lines. **(d)** Scatter plot illustrating all PUS7-dependant Ψ sites across the HeLa transcriptome. **(e)** Comparison of the modification levels of Ψ50 in cy-tRNA$^{Arg(CCT)}$ between WT and PUS7-KO cell lines. **(f)** Scatter plot illustrating all PUS1-dependant Ψ sites across the HeLa transcriptome. **(g,h)** Comparison of the modification levels of Ψ20 in mt-tRNA$^{Leu(UUR)}$ **(g)** and Ψ25 in mt-tRNA^{Asn} **(h)** between WT and PUS1-KO cell lines. **(i)** Comparison of the modification levels of PUS-dependent Ψ sites across cy-tRNAs (blue), mt-tRNAs (orange), and polyA-tailed RNA (green). For boxplots, boxes represent the 25th to 75th percentiles with a line at the median; whiskers correspond to 1.5 times the interquartile range (TRUB1: mt-tRNAs, *n* = 6; polyA-tailed RNA, *n* = 41; PUS7: cy-tRNAs, *n* = 69; polyA-tailed RNA, *n* = 22; PUS1: cy-tRNAs, *n* = 130; mt-tRNAs, *n* = 27; polyA-tailed RNA, *n* = 8).

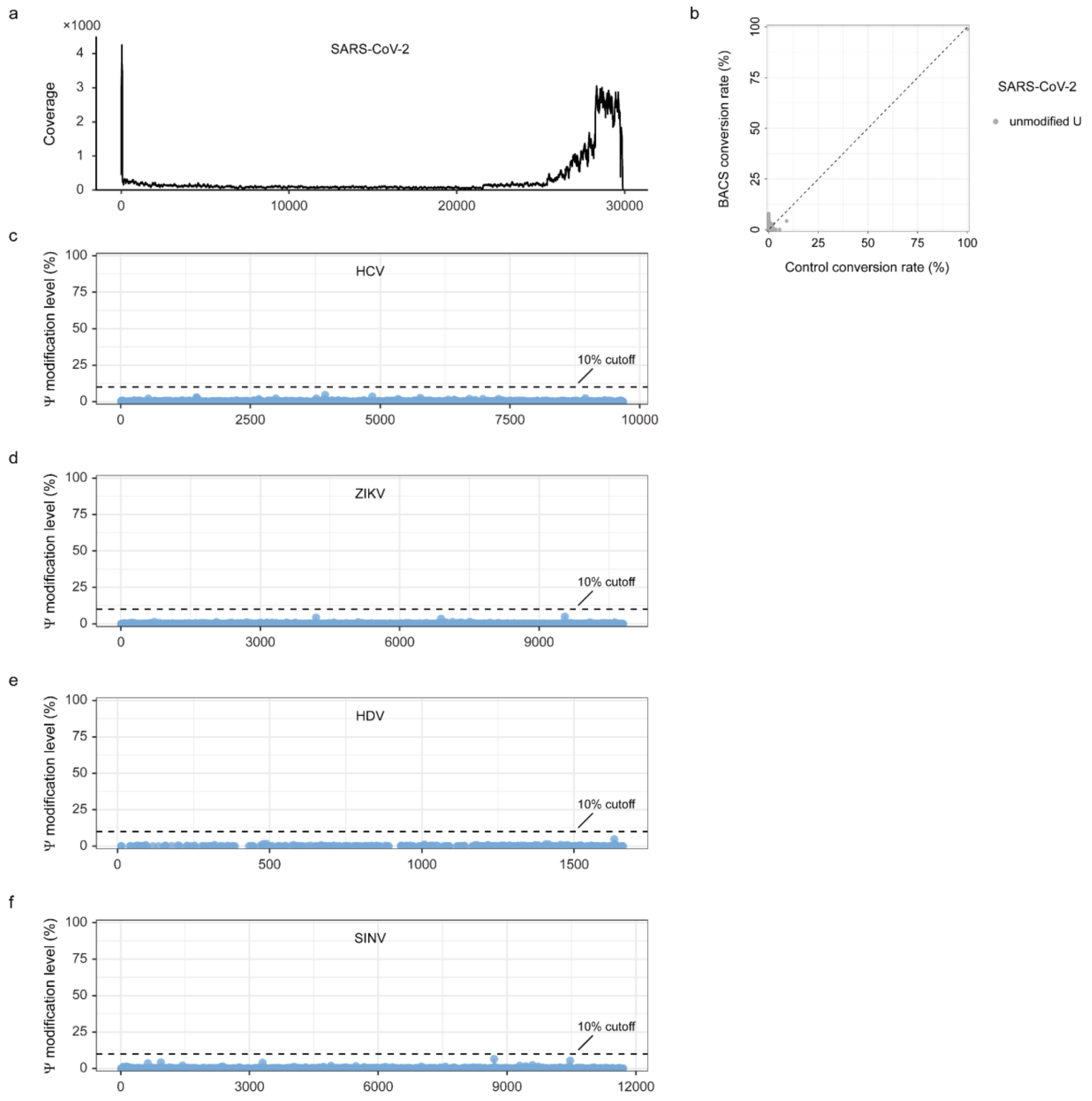

Supplementary Fig. 14 | Absence of Ψ in transcripts and genomes of RNA viruses. (a) Sequencing coverage of SARS-CoV-2 viral RNA. Similar to previous RNA-seq results[3], the majority of reads were mapped to the positive strand with a unique pattern at the 3'-end corresponding to the subgenomic RNAs. **(b)** Comparison of the conversion rates in SARS-CoV-2 viral RNA between BACS and control samples. **(c–f)** Ψ modification levels in HCV **(c)**, ZIKV **(d)**, HDV **(e)**, and SINV **(f)** viral RNAs.

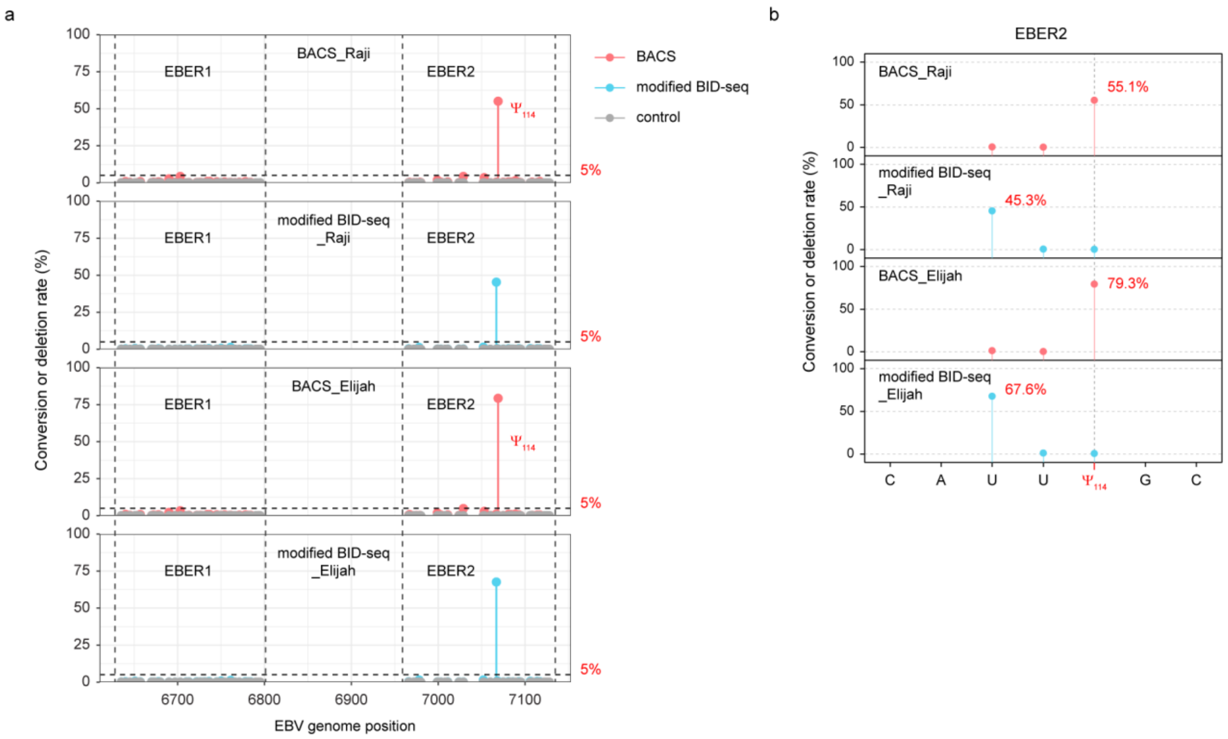

Supplementary Fig. 15 | Validation of Ψ_{114} site in EBER2. (a) Comparison of the conversion rates of BACS (pink) with the deletion rates of modified BID-seq (blue) for EBER1 and EBER2. **(b)** Comparison of the conversion rates of BACS (pink) with the deletion rates of modified BID-seq (blue) in the EBER2 Ψ_{114} region. Due to their deletion signals, BS-based methods cannot determine the exact position or identify the number of Ψ within consecutive uridine contexts. By default, the aligner will position the deletion at the 5'-most uridine.

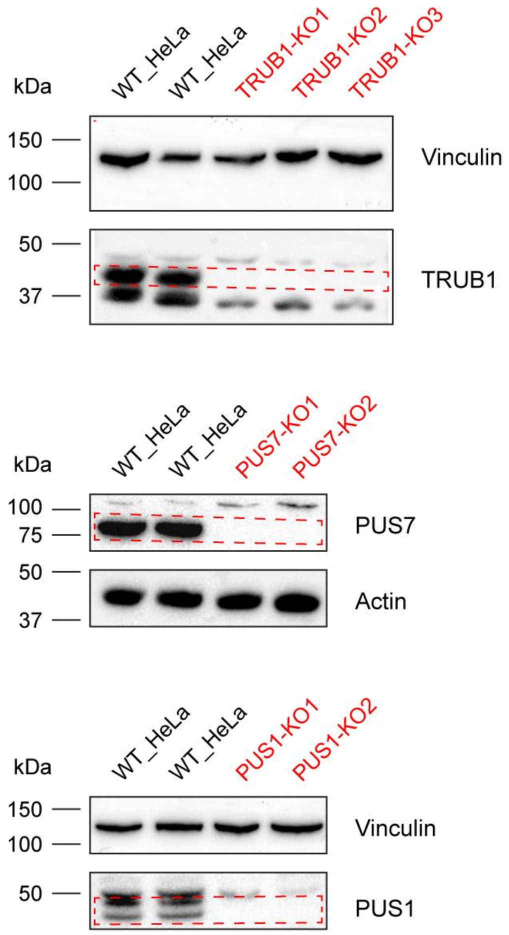

Supplementary Fig. 16 | Validation of TRUB1-KO, PUS7-KO, and PUS1-KO HeLa cell lines. Vinculin or β-actin was used as a loading control. Two independent experiments were performed with similar results.

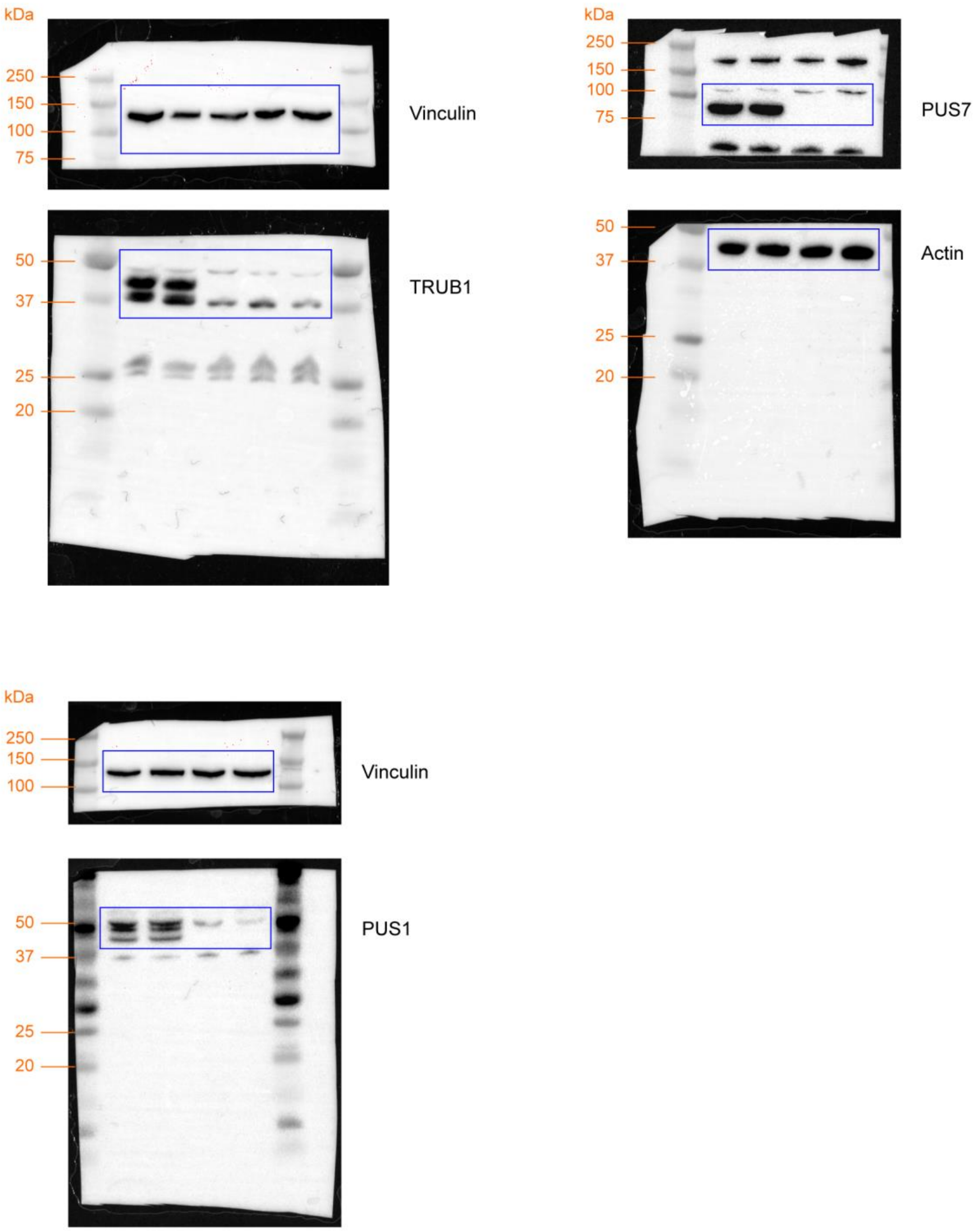

Supplementary Fig. 17 | Unprocessed western blots related to Supplementary Fig. 16.

Supplementary Table 1. RNA oligonucleotide sequences in this work.

Name	Sequence (5' to 3')	Source
for MALDI		
10mer U-ORN	UACUG**U**AGCU	IDT
10mer Ψ-ORN	UACUG**Ψ**AGCU	IDT
for mutation analysis		
72mer Ψ-ORN	GGGAGAACACACCACAACGAAACCAACG G**Ψ**ACAACAACAGAAA**Ψ**CGAGGACCGAAG CGAAGGCAAAGACAAC	*in vitro* transcription Pseudo-UTP, ATP, CTP, GTP
for UHPLC-MS/MS		
1.8-kb 10% Ψ-modified RNA	T7 *in vitro* transcription using linearized Fluc plasmid (NEB) as template	*in vitro* transcription 10% Pseudo-UTP, 90% UTP, ATP, CTP, GTP
spike-ins		
30mer NNUNN	AUGUCUCGACGU**NNUNN**GUUACAGUAC CGU	IDT
30mer NNΨNN	GCUUCAAGUUGA**NNΨNN**CAUCGCAAGU GCA	IDT

Supplementary Table 2. Compound-dependent UHPLC-MS/MS parameters used for nucleoside quantification. All the nucleosides were analyzed in the positive mode.

Compound	Precursor Ion (m/z)	Product Ion (m/z)	RT (min)	Delta RT (min)	CE (V)
Ψ+H	245	125	1.8	2.0	10.0
rC+H	244	112	2.3	2.0	10.0
rC+Na	266	134	2.3	2.0	10.0
rU+H	245	113	3.3	2.0	10.0
rG+H	284	152	8.1	2.0	10.0
rA+H	268	136	12.9	2.0	8.0

RT: retention time; CE: collision energy.

References:

1. Jorjani, H. et al. An updated human snoRNAome. *Nucleic Acids Res.* **44**, 5068–5082 (2016).
2. Safra, M., Nir, R., Farouq, D., Slutzkin, I. V. & Schwartz, S. TRUB1 is the predominant pseudouridine synthase acting on mammalian mRNA via a predictable and conserved code. *Genome Res.* **27**, 393–406 (2017).
3. Wing, P. A. C. et al. Hypoxia inducible factors regulate infectious SARS-CoV-2, epithelial damage and respiratory symptoms in a hamster COVID-19 model. *PLoS Pathog.* **18**, e1010807 (2022).
