# Supplementary material for: Absolute quantitative and base-resolution sequencing reveals comprehensive landscape of pseudouridine across the human transcriptome
Source: Nat Methods. 2024 Sep 30;21(11):2024–33. doi: 10.1038/s41592-024-02439-8 (PMC11541003; doi:10.1038/s41592-024-02439-8)
Supplement: Supplementary file 2 — Reporting Summary [file 41592_2024_2439_MOESM2_ESM.pdf]

nature portfolio

Corresponding author(s): Chun-Xiao Song

Last updated by author(s): Jul 30, 2024

Reporting Summary

Nature Portfolio wishes to improve the reproducibility of the work that we publish. This form provides structure for consistency and transparency in reporting. For further information on Nature Portfolio policies, see our Editorial Policies and the Editorial Policy Checklist.

Statistics

For all statistical analyses, confirm that the following items are present in the figure legend, table legend, main text, or Methods section.

n/a	Confirmed	
☐	☒	The exact sample size (*n*) for each experimental group/condition, given as a discrete number and unit of measurement
☐	☒	A statement on whether measurements were taken from distinct samples or whether the same sample was measured repeatedly
☐	☒	The statistical test(s) used AND whether they are one- or two-sided<br>*Only common tests should be described solely by name; describe more complex techniques in the Methods section.*
☐	☒	A description of all covariates tested
☐	☒	A description of any assumptions or corrections, such as tests of normality and adjustment for multiple comparisons
☐	☒	A full description of the statistical parameters including central tendency (e.g. means) or other basic estimates (e.g. regression coefficient) AND variation (e.g. standard deviation) or associated estimates of uncertainty (e.g. confidence intervals)
☐	☒	For null hypothesis testing, the test statistic (e.g. *F*, *t*, *r*) with confidence intervals, effect sizes, degrees of freedom and *P* value noted<br>*Give P values as exact values whenever suitable.*
☒	☐	For Bayesian analysis, information on the choice of priors and Markov chain Monte Carlo settings
☐	☒	For hierarchical and complex designs, identification of the appropriate level for tests and full reporting of outcomes
☐	☒	Estimates of effect sizes (e.g. Cohen's *d*, Pearson's *r*), indicating how they were calculated

Our web collection on statistics for biologists contains articles on many of the points above.

Software and code

Policy information about availability of computer code

Data collection	Illumina NextSeq 2000 and BaseSpace
Data analysis	Paired-end reads were download as FASTQ from Illumina BaseSpace. Statistical analyses were performed in R (v.4.0.3). The details are described in the Methods section. The analysis scripts are available at https://github.com/lkong888/bacs.<br>Cutadapt (v.4.2), UMI-tools (v.1.0.1), fastp (v.1.0.1), bowtie2 (v.2.4.4), STAR (v.2.7.9a), samtools (v.1.16.1), cpup (v.0.1.0), r2r (v.1.0.6), bedtools (v.2.30.0), featureCounts (v.1.6.4), enrichR (v.3.2), ggseqlogo (v.0.1), R (v.4.0.3 & v.4.3.1).

For manuscripts utilizing custom algorithms or software that are central to the research but not yet described in published literature, software must be made available to editors and reviewers. We strongly encourage code deposition in a community repository (e.g. GitHub). See the Nature Portfolio guidelines for submitting code & software for further information.

Data

Policy information about availability of data

All manuscripts must include a data availability statement. This statement should provide the following information, where applicable:

- Accession codes, unique identifiers, or web links for publicly available datasets
- A description of any restrictions on data availability
- For clinical datasets or third party data, please ensure that the statement adheres to our policy

All sequencing data are available at the GEO database (accession: GSE241849). All relevant additional data have been published with the manuscript, either as part

of the main text or in the supplement.

Reads were first mapped to human rRNA, snoRNA, and tRNA references:
Human rRNA sequences were downloaded from NCBI (NR_023363.1, NR_003285.3, NR_003286.4, NR_003287.4);
Human snoRNA sequences that belong to HGNC “Small nucleolar RNAs” gene group (https://www.genenames.org/) were downloaded from RefSeq (https://www.ncbi.nlm.nih.gov/refseq/);
High-confidence human tRNA sequences (hg38) were downloaded from GtRNAdb (https://gtrnadb.ucsc.edu/);
Unmapped reads were aligned to human genome (hg38) (https://genome.ucsc.edu/) with GENCODE v.43 (https://www.gencodegenes.org/).

For RNA viruses, the following reference genomes were used:
Severe acute respiratory syndrome coronavirus 2 isolate Wuhan-Hu-1 (NCBI, NC_045512.2);
Recombinant Hepatitis C virus J6(5’UTR-NS2)/JFH1 (NCBI, JF343782.1);
Zika virus isolate ZIKV/H. sapiens/Brazil/Natal/2015 (NCBI, NC_035889.1);
Hepatitis Delta Virus sequence from the pSVL(D3) plasmid (Addgene plasmid #29335) (https://www.addgene.org/29335/);
Sindbis virus (NCBI, NC_001547.1).

For EBV samples, reads were aligned to Epstein-Barr virus (EBV) genome, strain B95-8 (NCBI, V01555.2).

Related published data were downloaded from the GEO database: BID-seq for HeLa cells (GSE179798).

Human research participants

Policy information about studies involving human research participants and Sex and Gender in Research.

Reporting on sex and gender	n/a
Population characteristics	n/a
Recruitment	n/a
Ethics oversight	n/a

Note that full information on the approval of the study protocol must also be provided in the manuscript.

Field-specific reporting

Please select the one below that is the best fit for your research. If you are not sure, read the appropriate sections before making your selection.

☒ Life sciences ☐ Behavioural & social sciences ☐ Ecological, evolutionary & environmental sciences

For a reference copy of the document with all sections, see nature.com/documents/nr-reporting-summary-flat.pdf

Life sciences study design

All studies must disclose on these points even when the disclosure is negative.

Sample size	No statistical methods were used to predetermine the sample size. All sample sizes were determined based on our prior experiences on similar experiments and published studies (Liu et al., Nat. Biotechnol. 2019; Dai et al., Nat. Biotechnol. 2023).
Data exclusions	No data were excluded from the analyses.
Replication	Yes, as described in figure legends and Methods.
Randomization	Randomization was not relevant to this study. Controlling covariates was not necessary because experimental and control samples were processed in parallel.
Blinding	Blinding was not performed as this work is method development.

Reporting for specific materials, systems and methods

We require information from authors about some types of materials, experimental systems and methods used in many studies. Here, indicate whether each material, system or method listed is relevant to your study. If you are not sure if a list item applies to your research, read the appropriate section before selecting a response.

Materials & experimental systems

n/a	Involved in the study
☐	☒ Antibodies
☐	☒ Eukaryotic cell lines
☒	☐ Palaeontology and archaeology
☒	☐ Animals and other organisms
☒	☐ Clinical data
☒	☐ Dual use research of concern

Methods

n/a	Involved in the study
☒	☐ ChIP-seq
☒	☐ Flow cytometry
☒	☐ MRI-based neuroimaging

Antibodies

Antibodies used

TRUB1 antibody (Proteintech, #12520-1-AP) (1:1000);
PUS7 antibody (Abcam, #ab226257) (1:10000);
PUS1 antibody (Proteintech, #11512-1-AP) (1:1000);
β-Actin antibody (Cell Signaling Technology, #4967) (1:1000);
Vinculin antibody (Cell Signaling Technology, #13901) (1:3000).

Validation

TRUB1 antibody (Proteintech, #12520-1-AP): https://www.ptglab.com/products/TRUB1-Antibody-12520-1-AP.htm
Positive WB detected in HEK-293T cells, HepG2 cells
Positive IP detected in mouse liver tissue
Positive IHC detected in human kidney tissue, mouse testis tissue

PUS7 antibody (Abcam, #ab226257): https://www.abcam.com/products/primary-antibodies/pus7-antibody-ab226257.html
Positive control WB: HeLa, HEK-293T and Jurkat whole cell lysate
Cited in doi:10.1261/rna.078940.121

PUS1 antibody (Proteintech, #11512-1-AP): https://www.ptglab.com/products/PUS1-Antibody-11512-1-AP.htm
Positive WB detected in mouse lung tissue, HEK-293 cells, HeLa cells
Positive IHC detected in human ovary tumor tissue
Positive IF detected in MCF-7 cells
Cited in doi:10.1261/rna.078940.121

Eukaryotic cell lines

Policy information about cell lines and Sex and Gender in Research

Cell line source(s)

HeLa cells were gifted from Prof Peter J. Ratcliffe (University of Oxford) (originally obtained from ATCC, #CCL-2);
C666-1 cells were gifted from Dr Christopher Dawson (University of Warwick);
Raji and Elijah cells were gifted from Prof Paul Farrell (Imperial College London);
Vero-TMPRSS2 cells were obtained from NIBSC, #100978;
Calu-3 cells were gifted from Prof Nicole Zitzmann (University of Oxford) (originally obtained from ATCC, #HTB-55);
Vero cells were obtained from ATCC, #CCL-81;
Huh7.5 cells were gifted from Prof Charles Rice (Rockefeller University);
Huh7 cells were gifted from Prof Arvind Patel (University of Glasgow);
HepG2-NTCP cells were gifted from Prof Stefan Urban (University of Heidelberg);
BHK-21 cells were obtained from ATCC, #CCL-10;
A549 cells were obtained from ATCC, #CCL-185.

Authentication

C666-1 and Raji cell lines were authenticated by PCR assays with species-specific primers;
Vero cell line was authenticated by DNA barcoding;
Calu-3 and Huh7.5 cell lines were authenticated by STR profiling;
Other cell lines were not authenticated.

Mycoplasma contamination

All cell lines were negative for mycoplasma test.

Commonly misidentified lines
(See ICLAC register)

No commonly misidentified cell lines were used.
